# Using Satellite-Based Terrestrial Water Storage Data: A Review

**DOI:** 10.1007/s10712-022-09754-9

**Published:** 2023-01-13

**Authors:** Vincent Humphrey, Matthew Rodell, Annette Eicker

**Affiliations:** 1 Department of Geography, University of Zürich, Winterthurerstrasse 190, 8057 Zürich, Switzerland; 2 Institute for Atmospheric and Climate Science, ETH Zürich, Universitätstrasse 16, 8092 Zürich, Switzerland; 3 Earth Sciences Division, NASA Goddard Space Flight Center, Greenbelt, MD 20771 USA; 4 HafenCity University Hamburg, Überseeallee 16, 20457 Hamburg, Germany

**Keywords:** Terrestrial water storage, GRACE, Climatology, Hydrology, Remote sensing, Geodesy

## Abstract

Land water storage plays a key role for the Earth’s climate, natural ecosystems, and human activities. Since the launch of the first Gravity Recovery and Climate Experiment (GRACE) mission in 2002, spaceborne observations of changes in terrestrial water storage (TWS) have provided a unique, global perspective on natural and human-induced changes in freshwater resources. Even though they have become much used within the broader Earth system science community, space-based TWS datasets still incorporate important and case-specific limitations which may not always be clear to users not familiar with the underlying processing algorithms. Here, we provide an accessible and illustrated overview of the measurement concept, of the main available data products, and of some frequently encountered technical terms and concepts. We summarize concrete recommendations on how to use TWS data in combination with other hydrological or climatological datasets, and guidance on how to avoid possible pitfalls. Finally, we provide an overview of some of the main applications of GRACE TWS data in the fields of hydrology and climate science. This review is written with the intention of supporting future research and facilitating the use of satellite-based terrestrial water storage datasets in interdisciplinary contexts.

**Article Highlights**

- A summary of the GRACE satellite mission, the derived terrestrial water storage datasets, and the main technical terms and concepts
- A survey of the main challenges encountered in hydro-climate research when working with GRACE data and how to address them
- An overview of recent applications of GRACE TWS in water budget analyses, extremes monitoring, and Earth system modelling

## Introduction

Terrestrial water storage (TWS) is defined as the total amount of water stored on land. This includes any type of natural or artificial water reservoir, such as ground water, soil moisture, rivers, lakes, snowpack, glaciers, land ice, and water stored in biomass. TWS changes represent changes in terms of available freshwater resources which can have important impacts on both natural ecosystems and human activities. In response to the combined influences of natural climate variability and human interventions, TWS changes unfold over a wide range of temporal scales, ranging from short-lived extreme events caused by droughts and floods, to seasonal variability, inter-annual variability, and decadal trends related to, for instance, glacier mass loss or groundwater abstractions. As an integrator of changes in all water fluxes, TWS represents a key long-term memory variable for the water cycle and the Earth system in general, both responding to and feeding back to atmospheric and oceanic variability.

TWS will change in response to any imbalance between the main water fluxes that are precipitation (P), evapotranspiration (ET), and runoff (R). For this reason, it represents one of the key variables of the water budget (Eq. 1).

1 $$\frac{{{\text{dTWS}}}}{{{\text{d}}t}} = {\text{P}} - {\text{ET}} - {\text{R}}$$

TWS can also be expressed as the sum of all the potential water reservoirs.

2 $${\text{TWS}} = {\text{GW}} + {\text{SM}} + {\text{SWE}} + {\text{SW}} + {\text{LI}} + {\text{BW}}$$

Here, GW is the groundwater, SM is the soil moisture, SWE is the snow water equivalent, SW is the surface water, LI is the land ice, and BW is the biomass water.

Space-based observation of TWS changes debuted with the launch of the Gravity Recovery and Climate Experiment (GRACE) satellite mission in 2002. Before 2002, there were mainly two ways that TWS changes could be estimated. First, they can be estimated as the residual of the other observed water fluxes within the water budget equation (Eq. 1). Of course, this approach requires sufficiently accurate estimates of precipitation, evapotranspiration and runoff fluxes (Oki et al. 1995; Hirschi and Seneviratne 2017). The second approach is to estimate TWS as the sum of individually observed or modelled reservoirs (Eq. 2). In this case, one can either rely on estimates from a hydrological model or use extensive ground observations, provided these are available (Rodell and Famiglietti 2001). Depending on the climate type, certain of these TWS components may dominate TWS variability (e.g., snow and ice in polar and alpine regions) while others, like biomass water, can be safely ignored (Rodell et al. 2005; Getirana et al. 2017). Both of these approaches have serious limitations. In particular, these indirect estimates accumulate the measurement or estimation errors present in all the other terms, leading to potentially large uncertainties.

The GRACE mission provided the first direct observations of TWS changes at the continental scale (Wahr et al. 1998, 2004; Tapley et al. 2004). The mission’s goal was to measure very small variations of the Earth’s gravity field which are caused by the redistribution of masses over land, atmosphere, and oceans. In the next section, we will discuss the mission concept and data products in more detail. During the first years of the GRACE mission, obtaining precise estimates of TWS changes from the raw GRACE observations required developing and testing new retrieval algorithms and postprocessing strategies. Those techniques have since then made enormous progress, and GRACE-based TWS observations have reached a level of maturity enabling them to be used in a wide variety of applications, such as monitoring of freshwater resources and assimilation into numerical hydrological models (Tapley et al. 2019; Rodell et al. 2018). Following the success of the pioneering GRACE mission, gravimetric TWS remote sensing has entered a new phase where several successor missions are being proposed and implemented (Wiese et al. 2011; Elsaka et al. 2013; Pail et al. 2015; Haagmans et al. 2020; Flechtner et al. 2021). The objective is to both extend the existing data record and improve the spatiotemporal resolution of the final data products. In 2018, just one year after the initial GRACE mission ended, the successor GRACE Follow-On mission was successfully launched (Landerer et al. 2020). Some efforts have also been invested in producing publicly available long-term TWS records that cover the pre-GRACE era as well, mainly by means of data assimilation, machine learning, and statistical reconstructions (Kumar et al. 2016; Humphrey and Gudmundsson 2019; Li et al. 2021). Using only geodetic observations from satellite laser ranging (SLR), Löcher and Kusche (2020) have recently been able to extend the GRACE record back to 1992.

We provide in this article an overview of gravimetric TWS observations, of their usage, and of some of their applications. Section 2 provides a summary of the GRACE mission, of the main available hydrological data products, and of some frequently encountered concepts and issues that are part of the processing algorithms. Section 3 reviews some of the frequent challenges associated to using GRACE data products and offers guidance as to how to address them. Section 4 reviews a selection of applications that illustrate the use of space-based TWS observations in hydrology and Earth system sciences.

## GRACE Mission, Common Terms, and Data Products

### Mission Concept

The overall principle of the GRACE mission is to measure time-varying anomalies in the Earth’s gravity field, from which terrestrial water storage changes can be inferred (Tapley et al. 2004; Wahr et al. 2004). Because the water cycle and the global atmospheric circulation continuously redistribute enormous amounts of water mass around the globe, this causes local and extremely small changes in the Earth’s gravitational attraction. Such changes are on the order of 10^–8^ m s^−2^, which is a billion times smaller than the average value of *g* = 9.81 m s^−2^ (Wouters et al. 2014). Still, this is substantial enough to influence the orbits of satellites, especially when they have a relatively low altitude (i.e. initially 500 km for the GRACE satellites).

**Fig. 1:**
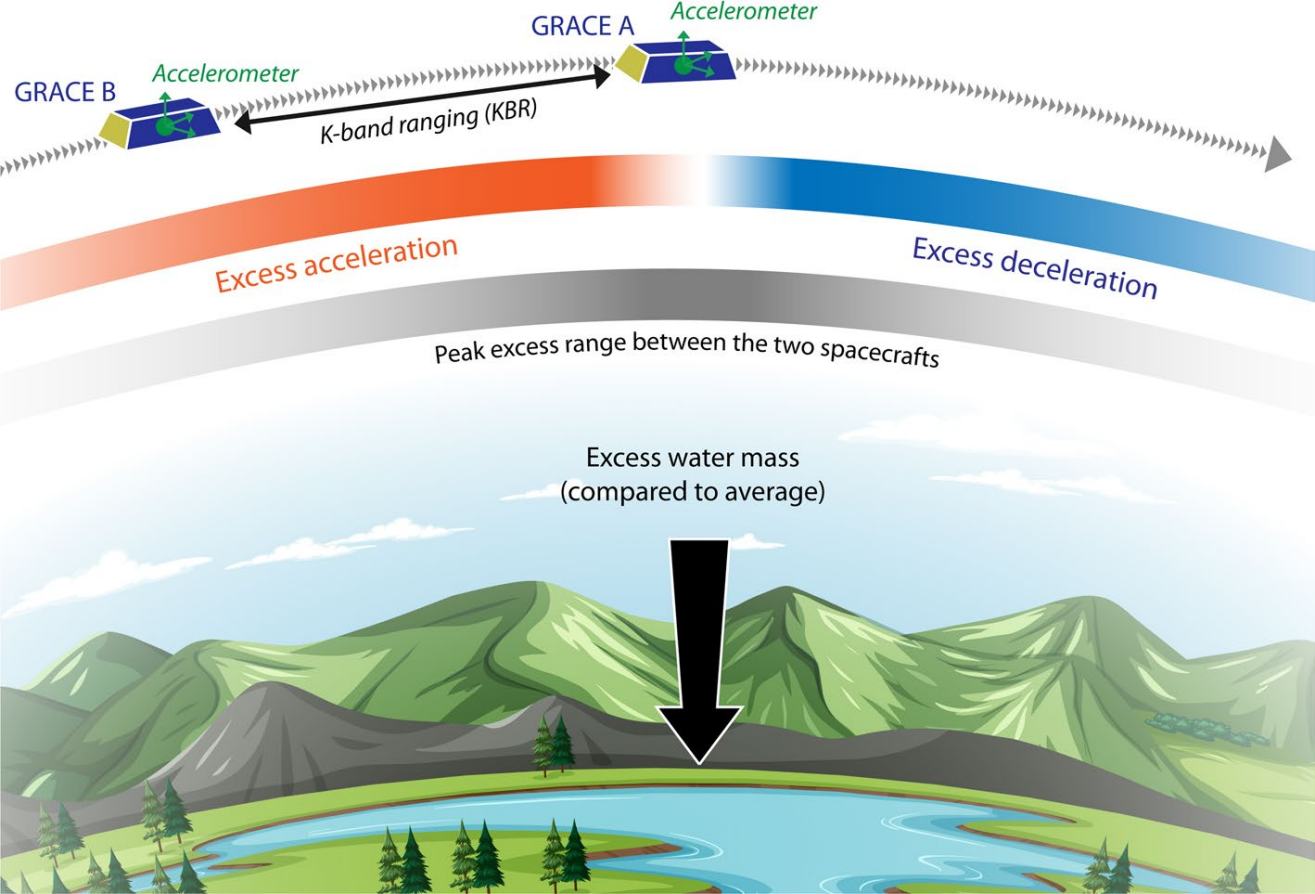
Measurement principle of the GRACE mission. The two GRACE satellites follow each other at an altitude of about 500 km, separated by a distance of about 220 km. When a region experiences an excess in mass, the gravitational attraction is locally stronger, causing the leading satellite to accelerate towards the positive mass anomaly. As a result the distance between the two spacecraft increases until the trailing satellite catches up. These variations in orbital behaviour are then used to infer time-dependent mass changes at the Earth’s surface. Background credit: freepik.com

A temporary excess in water mass, for instance wet soils caused by heavy rains, produces a temporarily stronger gravitational acceleration at that location. This in turn leads to minor variations in the behaviour of a spacecraft along its usual orbit (relative to what happens when the TWS is close its long-term mean). The spacecraft experiences a stronger gravitational pull when approaching a positive mass anomaly, leading to an along-track acceleration, followed by a deceleration immediately after passing the anomaly (Fig. 1). In situations where water mass is lacking (i.e. during a drought), the opposite behaviour is seen and along-track deceleration is followed by acceleration. Here, it becomes obvious that one key characteristic of GRACE is that it estimates mass anomalies relative to the long-term average gravity field. In other words, GRACE provides estimates of terrestrial water storage anomalies (${\text{TWSA}}={\text{TWS}}-\overline{{\text{TWS }}}$). Neither the total amount of TWS nor its long-term average ($\overline{\text{TWS}}$) can be measured with GRACE. To measure gravity field variations (and infer water mass changes from them), the first GRACE mission used a pair of twin satellites, with the first spacecraft flying about 220 km ahead of the other. The satellites were equipped with three-dimensional accelerometers to measure atmospheric drag and other non-gravitational accelerations, and the change in distance between the two spacecraft (on average 220 km apart) was continuously measured with a K-band microwave interferometer to a precision of about 1 μm per second. GRACE Follow-On can achieve nanometre precision with its laser ranging instrument. These measurements are needed to precisely monitor changes in the orbital trajectory of each satellite. The orbits are further constrained with observations from the on-board GPS receivers and star cameras.

**Fig. 2:**
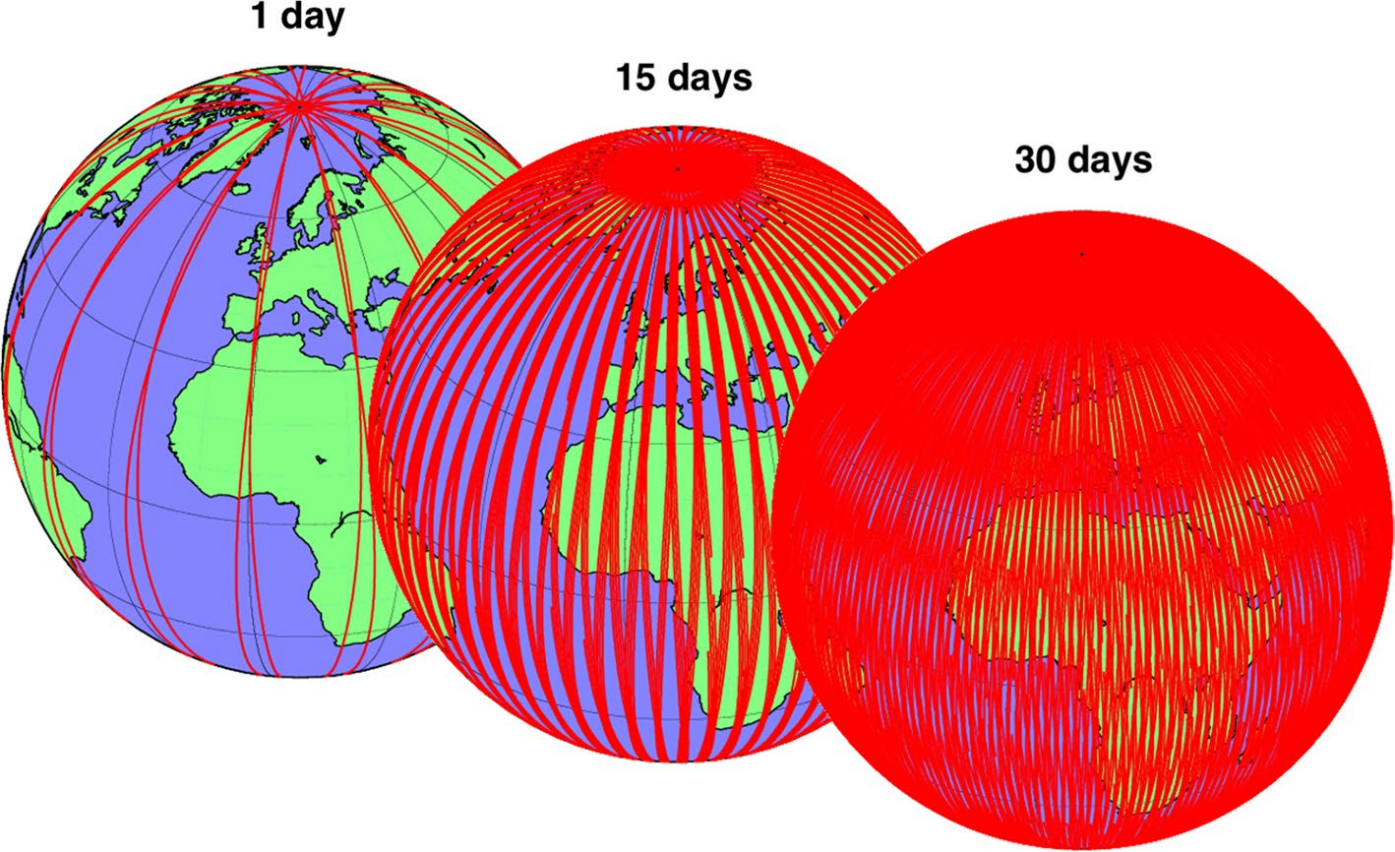
Satellite ground tracks of the GRACE mission (figure by Torsten Mayer-Gürr, Technische Universität Graz)

Of course, only substantial mass changes (typically occurring over a large spatial extent) can be recovered with this method. At the global scale (summing all land areas), the alternance between wet and dry seasons causes seasonal changes in global TWS (clearly visible in GRACE data) of about 6000 gigatons (equivalent to 6000 km^3^ of water) (Reager et al. 2016). This seasonal change in land water storage causes global sea level to fall and rise by about 17 mm every year as water is exchanged between land and oceans. GRACE satellites can also monitor TWS anomalies at smaller spatial scales, over areas as small as 150,000 km^2^ at mid-latitudes, and down to 50,000 km^2^ near the poles, where the satellite ground tracks are close together (Fig. 2; Rowlands et al. 2005; Swenson et al. 2006; Vishwakarma et al. 2018). As will be seen in the next sections, there are many factors which control the accuracy and resolution of GRACE data for a specific use case. Very often, choices in retrieval techniques and postprocessing algorithms may have different impacts on the interpretability of the data. Here, we provide an overview of the main concepts and terms that are commonly encountered when working with user-level GRACE data.

### Spherical harmonics

**Fig. 3:**
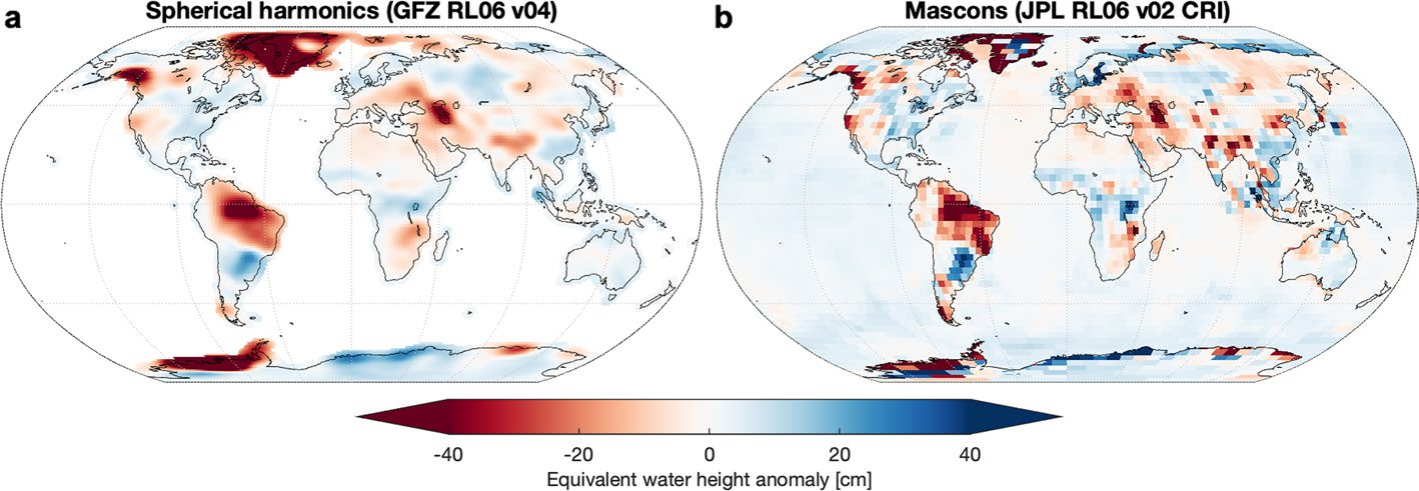
TWS anomalies for the month of December 2015, as recovered in two different GRACE products. **a** Spherical harmonic solution from the GeoForschungsZentrum (GFZ) and **b** mass concentration (mascon) solution from the Jet Propulsion Laboratory (JPL). Note that the mass anomalies are more concentrated but also blockier in the mascon solution. Both datasets were obtained from NASA’s GRACE Tellus website which provides a collection of data products suited for hydrological applications. In December 2015, there was a large drought over most of the Amazon basin which caused a significant negative TWS anomaly as illustrated here. *Source data*: **a** *GRC Tellus Land RL06 (GFZ)*, **b** *JPL RL06M Version 2.0* (as listed in Table 1)

Since the initial years of the GRACE mission, the standard approach to processing GRACE gravity data to infer mass change has been to represent the Earth’s gravity field using spherical harmonics. Spherical harmonics are functions that are particularly convenient for approximating the non-spherical shape of the Earth or its gravity field (Wahr et al. 1998). The so-called low-degree and low-order (Stokes) coefficients of these spherical harmonics represent large-scale features (like the Earth’s oblateness), while higher-degree and higher-order coefficients represent features at increasingly finer spatial scales. There is an upper limit to the number of coefficients which can be robustly estimated with a given set of GRACE measurements. If insufficient observations are available, high-degree coefficients will be very uncertain. With a month’s worth of GRACE measurements, it is generally accepted that one can reliably estimate spherical harmonic coefficients up to a degree and order of at least 60. This number puts a hard limit on the resulting spatial resolution of the mass change estimates, at about 330 × 330 km. Including a higher number of (less robust) coefficients with the aim of including more detailed spatial features is of course possible (e..g., at degree/order 120, resolution would be about 160 km); however, this will increase the uncertainty of the solution and lead to a much higher level of noise. As a result, more intense spatial filtering (smoothing) will be needed to reduce this noise, which often has the effect of damping the recovered TWS dynamics. In some of the GRACE literature, a resolution of about 3° × 3° (330 × 330 km) has been proposed as a possible compromise between having the highest possible resolution while still limiting the level of noise globally (Watkins et al. 2015). Thus, many of the available monthly GRACE hydrological products based on spherical harmonics are using solutions that are referred to as *up to* (or *truncated to*) degree and order 60 (Fig. 3a). Note that the trade-off between the number of observations used to constrain the solution and the achievable spatial resolution also makes it possible to generate weekly or even daily GRACE products albeit at lower spatial resolution or with reduced accuracy (Kurtenbach et al. 2012). Finally, we note that some of the low-degree coefficients (like C20, a coefficient related to the Earth’s oblateness) are not particularly well measured by GRACE and are often replaced with data from other sources like satellite laser ranging (SLR) (Loomis et al. 2020).

### Mass Concentration Blocks (Mascons)

The primary alternative to spherical harmonics for processing GRACE data and deriving mass anomalies is to use the so-called mass concentration blocks (abbreviated as *mascons*) (Rowlands et al. 2005, 2010). A mascon corresponds to a small, predefined region on the Earth’s surface, for instance a 3° rectangular grid cell (Fig. 3b). The approach applies time-dependent mascon parameters to adjust a static (forward modelled) gravity field so that it matches the time-dependent mass surpluses or deficits. This procedure is different from spherical harmonics in the sense that a mascon is not a representation of the global gravity field, but instead serves to quantify a local mass anomaly. The static gravity field is typically a spherical harmonic solution based on several years of GRACE data that acts as a high-resolution and low-noise reference. An advantage of having location-dependent mascon parameters is that they can be more easily constrained based on a priori information, thus providing some improvement in terms of the achievable spatial resolution, especially over polar regions which are more densely sampled by GRACE’s ground tracks (Luthcke et al. 2006). The estimation of mascon parameters can be improved by using as an additional constraint an expected average pattern of covariances or variances between neighbouring mascons (taken for instance from a hydrological model or a previous GRACE solution) (Watkins et al. 2015; Save et al. 2016). An additional potential constraint is to implement temporal correlation in the mascon parameters, taking advantage of the fact that many large-scale mass changes unfold relatively slowly and are thus autocorrelated in time. In contrast, spherical harmonic solutions are global in nature and each monthly solution is usually independent from the next. Note that spatial and/or temporal constraints can also be introduced in spherical harmonics, as in Kurtenbach et al. (2012) or in Save et al. (2012).

For hydrological applications, key advantages of constrained mascon products compared to unconstrained spherical harmonics are that the original magnitudes of the TWS signals are better recovered and the TWS changes (especially along coastlines) are better resolved spatially (Scanlon et al. 2016) (also see Fig. 3). Mascon products are also easier to use in general as they do not need to be spatially filtered (smoothed) during postprocessing. This is because some (geophysically motivated) spatial information is explicitly guiding the retrieval of the mascon solution, thus greatly reducing the spatial noise patterns. While such constraints are effective in reducing the noise, the number of observations necessary to compute a solution still places an upper limit on the achievable maximum spatial resolution (see the Section 2.2 on ‘Spherical harmonics'). Thus, while some centres provide mascons that have a relatively fine spatial resolution, for instance at 1° (110 × 110 km) grid, the effective spatial resolution remains closer to 300 × 300 km (Save et al. 2016).

### De-aliasing

**Fig. 4:**
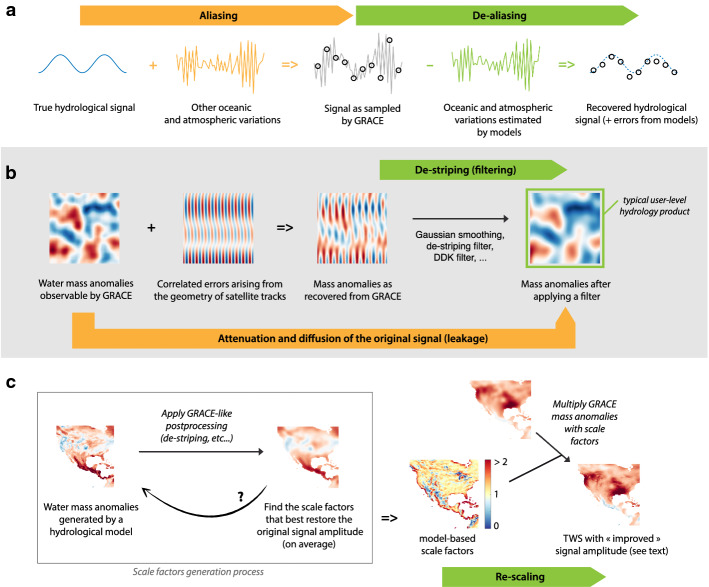
Illustration of some of the frequently encountered concepts in GRACE processing. **a** Aliasing refers to the contamination of a low-frequency signal, here the hydrological variability, by an undersampled high-frequency signal, here tidal and non-tidal effects on the geopotential. De-aliasing refers to the (imperfect) removal of aliasing using model estimates of the high-frequency signals. **b** Simplified representation of the correlated errors affecting the mass anomalies recovered by GRACE and the use of filtering techniques to remove them. An unwanted effect of filtering is that it also attenuates and mixes up the true geophysical signals, leading to a bias in the retrieved TWS amplitude and a leakage of TWS signals between neighbouring regions. **c** Generation process and usage of the model-based scale factors designed to correct for biases in GRACE TWS when computing regional averages at a larger scale

Temporal changes in the Earth’s gravity field are not only happening because of terrestrial water storage variations, but also due to many other processes. For instance, the atmospheric and oceanic circulations cause gravity field changes at temporal scales of a few hours to a few days (Dobslaw et al. 2013). Because GRACE observations are sensitive to these processes as well, the variability caused by atmospheric and oceanic processes is said to be *aliased* onto the GRACE measurements (Fig. 4a). In other words, the gravity signal associated with land hydrological processes is convolved with gravity signals resulting from higher-frequency processes. In order to isolate the hydrological effects, the high-frequency signals need to be estimated with observation-driven atmospheric reanalysis (i.e. weather models) and then subtracted from the GRACE measurements. This process is called *de-aliasing*. Several de-aliasing products are available, such as the AOD1B product from the German GeoForschungsZentrum (GFZ) (Dobslaw et al. 2017). For end-users of GRACE hydrology data products, the choice of the de-aliasing method is rarely an issue because this step is performed during the generation of TWS anomaly fields. However, it is worth knowing that errors in the atmospheric and oceanic circulation models, together with spatial and temporal undersampling of the gravity field changes associated with those circulations (which complicates their removal) do contribute a large fraction of the errors in retrieved TWS anomalies (Han et al. 2004; Seo et al. 2008). In addition to reducing aliasing errors, de-aliasing also implicitly removes the contribution of the atmosphere to monthly mass changes.

### De-striping and Filtering

A long-standing issue with GRACE data processing has been the presence of unphysical north–south-oriented stripes in maps of mass anomalies (e.g., Wouters et al. 2014). The origin of these stripes lies mainly in the polar configuration of the GRACE orbits and the along-track orientation of the two satellites themselves, which causes north–south gradients to be much better observed than east–west ones. Simulation studies have shown that this orbit configuration tends to magnify inaccuracies in the de-aliasing products, causing striping in the gravity solutions (Seo et al. 2008). Recent work by Peidou and Pagiatakis (2020) has also shown that striping may result from sub-Nyquist artefacts caused by latitudinal oversampling of the low-frequency gravitational signal of the geoid. In addition to stripes, measurements errors and noise from many other sources also cause residual errors which contaminate the maps of TWS anomalies obtained from raw GRACE solutions.

Many approaches have been proposed to reduce striping patterns as well as other errors in the most efficient way. Originally, large-scale (i.e. up to 1000 km) Gaussian filtering was applied to the data, effectively removing the noise, but with the negative consequence that many real smaller-scale features would be entirely smoothed out (Wouters et al. 2014). This posed a significant challenge because evaluating the first GRACE TWS anomalies against hydrological models required that the model data undergo a comparable filtering (Schmidt et al. 2006). More advanced techniques take advantage of the anisotropy of the striping patterns to optimally remove the noise while still retaining most of the real spatial patterns (Swenson and Wahr 2006b; Kusche 2007). The objective of these de-striping and filtering algorithms has generally been to improve gridded GRACE hydrology products or region-specific time series that are based on spherical harmonics and make them more easily comparable to other model-based or observational datasets. For mascon solutions, this problem is much less prominent because the a priori constraints used in the solutions explicitly mitigate the noise. Thus, it is not necessary to apply de-striping or filtering during mascon postprocessing.

### Leakage

Due to the truncation of spherical harmonics, the de-striping, filtering, and/or constraining processes, gridded maps of TWS anomalies have a very high degree of spatial autocorrelation which is, for a large part, not representative of the real TWS anomaly patterns. The spatial smoothing and filtering inherent to GRACE processing algorithms means that the TWS anomaly estimated at any given grid cell also incorporates TWS signals occurring in the neighbouring region, outside the area of interest. The consequence is that real TWS anomaly patterns may be distorted and signal amplitudes will be generally damped. In the GRACE community, this effect is usually referred to as signal leakage. Figure 4b illustrates it for the general case of spherical harmonics postprocessing. Some authors prefer to use the term *leakage* to describe the contamination by external signals and use the term *bias* to describe signal damping (Klees et al. 2007). Other authors use the term leakage to describe both effects (Swenson and Wahr 2002).

Signal *bias* and *leakage* are problematic for hydrological applications which attempt to isolate the TWS anomalies occurring within a specific region like a watershed or a large aquifer. It is even worse when damped TWS changes are combined or compared with fluxes in a water budget analysis. Various approaches have been proposed to address this issue, which affects spherical harmonic solutions more than mascon solutions. If the region of interest is precisely defined, a specifically optimized averaging kernel (or function) can be used to minimize the contamination by TWS signals that are located outside of the region of interest (Swenson and Wahr 2002). These averaging kernels are also used to calculate the expected signal attenuation and estimate a multiplicative correction factor which is used to restore (or rescale) the signal amplitude (Velicogna and Wahr 2006; Rodell et al. 2009), thus mitigating signal bias (though not leakage). In some cases, hydrological model data may also be used to estimate and remove the leakage contribution of TWS signals outside of the area of interest (Swenson and Wahr 2007). For the case of signal leakage across coastlines (i.e. between land and ocean mass changes), specific approaches have been developed to further reduce leakage and better separate the land and ocean mass contributions (Chen et al. 2015; Wiese et al. 2016; Tregoning et al. 2022). However, leakage and bias correction techniques are in general too cumbersome to implement for hydrologists, glaciogists, or other end-users who are not familiar with GRACE data processing and geodetic techniques. To spare users from having to estimate the averaging kernel and correction factors specific to their region of interest, gridded scale factors were developed to rescale the gridded GRACE products directly, thus providing a relatively straightforward way to address at least signal bias.

### Gridded Scale Factors

Several GRACE data products intended for hydrological applications provide an accompanying set of scale factors as a separate grid. These scale factors are intended to counteract damping of the TWS signal caused by the GRACE processing algorithms and restore the amplitude of TWS anomalies to a more accurate level (Landerer and Swenson 2012). The scale factors are calculated by applying GRACE processing algorithms to TWS data from a hydrological model. A comparison between the original model data and the postprocessed model data is used to diagnose the effect of postprocessing on signal amplitudes. The scale factors are then optimized to restore the original amplitude of the model data after it has been subjected to GRACE processing algorithms. This means that scale factors are estimated independently of GRACE data. In theory, the approach does not taint the resulting scaled GRACE TWS data with modelled hydrology. However, any inaccuracies in the patterns and magnitudes of the model simulated TWS will cause the scale factors to be suboptimal. In the example of Fig. 4c, one can see how GRACE processing algorithms have blurred most of the small scale features present in the original model simulation. Coastal regions have a mixed contribution from both land and ocean signals, such that their signal amplitudes are generally reduced. In regions with sharp transitions, grid points that had the strongest TWS variability experience a damping while grid points that had little TWS variability see an enhancement. The multiplicative scale factors are designed to optimally restore the TWS variance to its original level (typically by minimizing the least-square difference between the original and filtered hydrological model data) (Landerer and Swenson 2012). Scale factors higher (lower) than 1 indicate regions where GRACE TWS anomalies have been damped (magnified) by the postprocessing. The correction is applied by multiplying the GRACE TWS grids with the scale factors. It is worth noting that scale factors only address the average *bias* in TWS amplitude; however, they do not change the shape of the TWS time series. As a result, some *leakage* effects are still present even in scaled GRACE TWS products. In other words, only the variance is corrected in scaled GRACE TWS products, but the actual shape of the TWS time series or its correlation with neighbouring grid points (for example) is still affected by the spatial mixing inherent to GRACE data processing (mainly in a radius of 150 km around the point of interest). Users have to determine on a case by case basis whether *leakage* is adequately resolved by scaling. Further, it is crucial for users to recognize that, with or without scaling, time series of TWS anomalies computed from a gridded GRACE TWS data product are only meaningful when they are averaged over a sufficiently large region, typically at least 200,000 km^2^. In other words, due to leakage and despite the use of scale factors, a TWS time series from a single grid cell is not representative of conditions in that cell alone; it contains a substantial amount of signal from the larger surrounding region. Neighbouring grid cells should never be considered as statistically independent samples.

For the case of mascons, the role played by the scale factors is somewhat different. For example, the mascons provided by the Jet Propulsion Laboratory (JPL) have a relatively coarse resolution of 3° equal-area spherical caps and require no further averaging over a larger region if used in that form (Watkins et al. 2015). Because the 3° JPL mascons are provided on a 0.5° grid, all grid points belonging to a given mascon are identical and correspond to the TWS average of that mascon. This is what causes the blocky structure when the JPL mascons are mapped (Fig. 3b). Scale factors may be used to obtain a more realistic spatial distribution of mass within each given mascon, thus facilitating the delineation of study regions. In summary, hydrological model data are used to estimate how TWS variability should be spatially distributed (on average) within each mascon. Applying the (multiplicative) scale factors redistributes the amplitude of mass changes within the mascon without altering the mascon-level average. Only the variance of the individual grid points is scaled, but the temporal TWS dynamics remain identical over the whole 3° mascon. Using these scale factors massively improves the quality of regional- or basin-scale averages computed from the JPL mascons grids (Wiese et al. 2016).

There are a few limitations to the use of scale factors. The first limitation is that they were developed for the purpose of correcting the amplitude of the TWS features which explain the most variance in the TWS time series. In most cases, this means restoring the amplitude of the TWS seasonal cycle (Humphrey et al. 2016). However, applying the scale factors changes the amplitude of all temporal features, including that of the long-term trends, for example. It has been demonstrated that the seasonal cycle and the long-term trend may actually require different scale factors (Landerer and Swenson 2012). As a result, one should not expect that applying the standard scale factors will yield more accurate TWS trends at high spatial resolution (even though trend scaling is likely to be necessary). A second limitation is that they are based on hydrological model output. A comparative study by Long et al. (2015) showed that different hydrological models yield different scale factors, especially over arid, semi-arid, and intensively irrigated areas. Scale factors derived over temperate or humid regions were found to be more consistent. The role of scale factors is also larger for TWS averages computed over small basins (< 200,000 km^2^) compared to larger ones. More generally, it is important to understand that any spatial information gained by re-scaling the GRACE TWS grids is based on a hydrological model’s representation of the average distribution of TWS variability within the region. As scale factors are only intended to restore signal amplitude, they do not provide a correction for signal leakage (i.e. the potential mixing of heterogeneous TWS signals across space). This is important to consider when comparing maps of GRACE and modelled TWS, or when attempting to close the water budget in combination with other data products. In all cases, it is critical to take into account the effective spatial resolution of GRACE TWS data. One possibility is to first average the other data products to a resolution comparable with GRACE TWS grids. For instance, using the non-scaled 3° JPL mascons, this can be done by first aggregating higher-resolution datasets over the footprint of individual 3° mascons before making a comparison. With scaled spherical harmonics or scaled mascons, the comparison can be made using averages over sufficiently large spatial domains (e.g., > 200,000 km^2^ at low to mid-latitudes).

### Glacial Isostatic Adjustment

Glacial isostatic adjustment (GIA) refers to the rise and fall of land masses in response to the spatial redistribution of land ice and ocean masses over glacial cycles. These processes cause secular trends in the gravitational potential which are also measured by GRACE (Steffen et al. 2009; A et al. 2013). Thus, most GRACE products intended for hydrological applications have had the effects of GIA removed using models of the glaciation history and mantle viscosity. For instance, the ICE6G-D model of Peltier et al. (2015) is one of the GIA models which has been often used. In hydrological applications, this correction is essential for the computation of long-term TWS trends (Lambert et al. 2013; Rodell et al. 2018). If the effect of GIA is not properly removed, it will lead to spurious estimates of long-term TWS trends, in particular over previously ice-covered regions like the area around Hudson’s Bay in North America and Northern Europe. The uncertainty related to the GIA correction should be considered when making detailed analyses of the long-term TWS trends over these regions. For instance, one possibility is to compare GRACE TWS products which used similar processing methods but different GIA correction models.

## Understanding and Using GRACE TWS Data

### TWS Anomalies: Meaning and Units

Most GRACE or GRACE-FO Level-3 data products destined for hydrological applications provide monthly TWS changes as grids of anomalies (Table 1). An anomaly is a deviation from the mean state. This means that even though TWS is commonly defined as the sum of all water storages (Eq. 2), space-based gravimetry can in fact only measure its anomaly relative to a long-term mean (also see Fig. 1). These anomalies are obtained by subtracting a long-term average (also called baseline) gravity field ($\text{TWSA}=\text{TWS}-\overline{\text{TWS}}$). This point is often a subject of confusion to inexperienced users who expect GRACE data to provide them with the total water mass amount. In the literature, it is frequent to see GRACE data labelled as either TWS or TWS anomalies (TWSA), even though both terms refer to anomalies in this context because absolute TWS cannot be measured with GRACE. When comparing TWS anomalies gathered from different GRACE products or from other datasets (like hydrological model data), it is important to ensure that the anomalies are relative to the same baseline. If this is not the case or if the baseline is unknown, a time interval common to all TWS time series can be arbitrarily chosen and the anomalies re-calculated by subtracting from each dataset its own long-term average computed over that common time interval.

**Table 1:** Non-exhaustive list and characteristics of regularly updated spherical harmonics and mascon TWS products

Product name	Temporal resolution	Effective spatial resolution*	Grid	Technique	Serial correlation of solutions	GIA correction	Filtering	Ocean/land leakage correction	Gridded uncertainty estimates	Scale factors available	URL
JPL RL06M Version 2.1	Monthly	3°	0.5°	3° equal-area mass concentration blocks	Yes, part of the retrieval process	ICE6G-D	Not necessary	Yes (CRI)	Yes time-dependent	Yes based on CLM4	1
GRC Tellus Land RL06.1	Monthly	3° (up to 2° at latitudes > 70)	1°	Spherical harmonics solutions from three different centres	No, individual months are independent	ICE6G-D	Already applied	None	Yes time-dependent	Yes based on CLM4	2
CSR RL06.1 Mascon solutions	Monthly	3° (up to 2° at latitudes > 70)	0.25°	1° near equal-area mass concentration blocks	No, individual months are independent	ICE6G-D	Not necessary	Yes	None	No	3
GSFC long-term trend mascons	Single estimate	1°	Non gridded	1° equal-area mass concentration blocks	Not applicable	ICE6G-D	Not necessary	None	None	No	4
ITSG-Grace2018	Monthly and daily	3° for monthly (up to 2° at latitudes > 70) and 4.5° for daily	Needs to be defined by user	Spherical harmonics	Independent months, Kalman smoothing for daily solutions	Needs to be applied by user	Needs to be applied by user	None	None	No	5

*This is a global recommendation. Spatial resolution might be higher (lower) in favourable (unfavourable) cases, see, for example, Vishwakarma et al. (2018)

1 https://grace.jpl.nasa.gov/

2 https://grace.jpl.nasa.gov/

3 http://www2.csr.utexas.edu/grace/RL06_mascons.html

4 https://earth.gsfc.nasa.gov/index.php/geo/data/grace-mascons

5 https://www.tugraz.at/institute/ifg/downloads/gravity-field-models/itsg-grace2018

In GRACE or GRACE-FO hydrology products, TWS is usually reported in metres, centimetres or millimetres. This indicates the quantity of excess (or lacking) water mass that is needed at the surface of the Earth’s ellipsoid to best explain the observed gravity field anomaly. The terms of *equivalent water height* or *equivalent water thickness* may often be used in these products and reflect this representation of TWS as a single layer of water with a given height. Although TWS anomalies may not lie exactly at the surface of the ellipsoid (i.e. groundwater or glacier changes may happen at an altitude above or below that reference), this nuance has in practice no impact for hydrological applications. In principle, no unit correction is needed to use GRACE TWS data in hydrological applications. For some basin-scale or regional-scale studies, it may be desirable to obtain the water mass or volume (for instance, in gigatons or km^3^ of water). In this case, a common practice is to compute the mean TWS anomaly over the whole region of interest from the gridded TWS data and then multiply this value by the area of the region of interest.

### Grid size Versus Spatial Resolution

GRACE hydrology products are usually provided to end-users on a geographic grid with a resolution that is much finer than the actual ‘operational’ resolution of the GRACE satellites. For instance, the monthly JPL GRACE Mascons product is provided on a 0.5° grid (≈ 55 km), even though the actual size of an individual mass concentration block is 3° (≈ 330 km) (Scanlon et al. 2016). Similarly, the monthly GRACE Tellus spherical harmonics solutions are provided on a 1° grid (≈ 110 km), even though their actual resolution is also of about 3°. As explained in Sect. 2.2, the actual resolution of GRACE data depends on the number of observations available to calculate the gravity field estimate, as well as on what is considered an acceptable signal-to-noise ratio (Swenson et al. 2003). For monthly products, this usually leads to a spatial resolution of about 330 km, while for weekly or daily products, the resolution is of about 600–1000 km (Kurtenbach et al. 2012; Croteau et al. 2020). The achievable spatial resolution is improved at very high latitudes, where the density of GRACE ground tracks is much higher due to the polar orbit of the satellites (Fig. 2). In addition, in cases where a strong TWS signal originates from a confined area surrounded by little to no TWS variability, the achievable resolution (for a given signal-to-noise ratio) may also be higher (Vishwakarma et al. 2018).

Providing TWS anomalies on an oversampled grid makes it easier for end-users to aggregate GRACE data to their preferred format, even though the grid size does not reflect the effective resolution of GRACE. For example, regional averages may be calculated based on a user-defined watershed or aquifer mask which will be better represented on a 0.5° grid. However, regional TWS signals averaged over a small region (< 200,000 km^2^) or a large but very elongated region are likely to include a significant contribution from areas located outside of the region of interest. This may complicate attempts to close the water balance equation or compare TWS anomalies against data from other sources. To some extent, this problem can be mitigated by using the provided set of scale factors (but see Sect. 2.7 for a discussion).

### Comparability with Other Datasets

Because the effective spatial resolution of GRACE TWS is often coarser compared to other observational or model-based data products, these ancillary datasets usually need to be spatially aggregated or processed before they can be properly compared to GRACE. If this is neglected, some of the disagreement or phase difference between the two datasets may be interpreted as real, when it is in fact an artefact of the data processing. This is particularly critical when comparing GRACE TWS against hydrological model data. Since the first GRACE observations became available, there have been various ways of improving the comparability with other datasets. One option is to apply to the ancillary data the same processing and postprocessing algorithms used to obtain the GRACE TWS estimates. This was done for instance in early comparisons between GRACE TWS and hydrological model TWS. The hydrological model data are converted to spherical harmonics, truncated at a given degree–order, and any filters and transformations (de-striping, Gaussian filter, scale factors) are subsequently applied to the model data (e.g., Schmidt et al. 2006; Landerer and Swenson 2012; Döll et al. 2014). Similar procedures have been applied for instance to satellite soil moisture data or meteorological datasets (e.g., Abelen and Seitz 2013; Humphrey et al. 2016). A second, and often more practical option, is to average both GRACE TWS and the ancillary data over a sufficiently large area like a water basin (at least > 100,000 km^2^ and usually > 200,000 km^2^). This is most easily done using a basin mask and *scaled* gridded GRACE TWS data (e.g., Scanlon et al. 2018). The comparison is then conducted at the level of these regional averages. Note that it is also possible to simply use a coarser grid size instead of specific basin masks, for instance Jensen et al. (2019) have compared GRACE long-term trends with CMIP5 climate model data at a common resolution of 2°. A third option is to use the JPL mascon products, which are defined over 3° equal-area spherical caps, and average the ancillary data over the footprint of each mascon (e.g., Humphrey et al. 2018; Levine et al. 2019). The comparison is then conducted at the level of (unscaled) mascons. Finally, there are also cases where no transformation is applied to the higher-resolution ancillary data and comparisons are conducted directly at the GRACE grid resolution (e.g., Yang et al. 2014; Tian et al. 2018). While not recommended, this may be acceptable but only as long as scale factors have been applied to GRACE data and that the analysis does not treat neighbouring grid points as independent observations and does not aim to contrast the behaviour of neighbouring grid points.

### Irregular Months and Missing Months

**Fig. 5:**
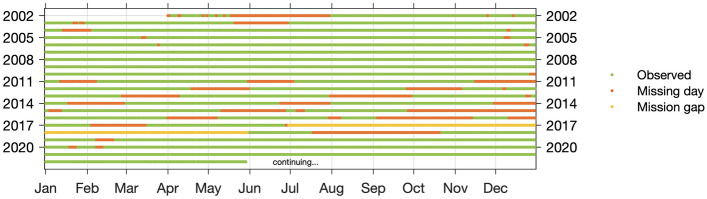
GRACE and GRACE Follow-On data coverage until June 2022 based on the GRC Tellus website (https://grace.jpl.nasa.gov). Solutions provided by some centres may have additional missing days

As explained in Sect. 2.2* Spherical harmonics*, about a month of GRACE observations are required in order to robustly estimate the gravity field at a spatial resolution of 3°. For some months, some observations may be missing due to maintenance operations, instrument issues, power interruptions, etc. If there are no sufficient observations to produce an average for a given month, additional data is often ‘borrowed’ from the previous or next month. As a result, GRACE monthly data have irregular monthly averages which do not necessarily correspond to normal months. For example, the December 2015 map shown in Fig. 3 actually uses observations gathered over the period from December 10^th^ 2015 to January 3^rd^ 2016. Users need to determine themselves if this represents a critical problem which they need to account for in their comparisons, for instance by computing monthly averages that accurately match the days used in the GRACE solutions. In addition, there are a number of missing months due to battery management over the period 2011–2017, as well as an 11-month gap from July 2017 to May 2018 between GRACE and GRACE Follow-On. A summary of data coverage by year is shown in Fig. 5.

### Integration with the Water Budget

**Fig. 6:**
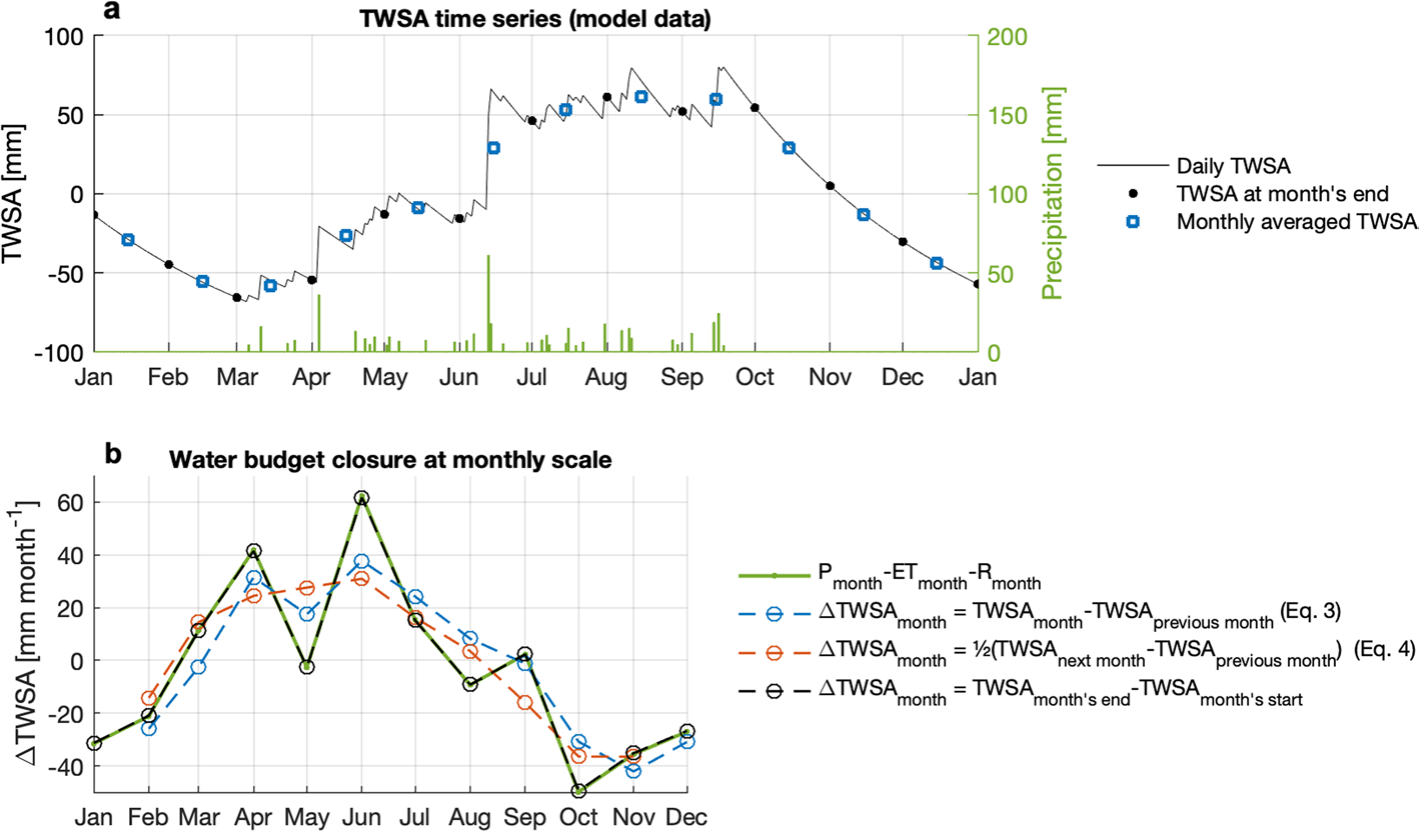
**a** Illustrative TWS anomaly time series generated with a simplified hydrological model. **b** Water budget closure at monthly scale between the monthly sum of water fluxes (*green*), and monthly TWS changes estimated with different approaches: backwards differences of monthly TWS averages (*blue*), centred differences of monthly TWS averages (*orange*), and the actual monthly storage change calculated from the daily TWS time series (*black*)

As mentioned earlier, GRACE TWS provides monthly anomalies relative to the long-term TWS average at each location. Using these values in combination with the water budget equation (Eq. 1) can be particularly challenging (Rodell et al. 2004), especially when using monthly precipitation, evapotranspiration, or runoff data (for instance because daily estimates of these fluxes are not available). In the water budget equation, the storage change noted in Eq. 1 corresponds to the difference between the start and the end of the considered time period. However, GRACE never provides such instantaneous estimates of TWS, only temporal averages taken over the whole time period (Fig. 6a). As a result, differentiating monthly GRACE data only provides an *approximate* estimate of $\text{dTWS}/dt$ (Rodell et al. 2004; Swenson and Wahr 2006a). This can be easily demonstrated for various difference operators and synthetic ‘toy’ model data (Fig. 6b).

Swenson and Wahr (2006a) originally evaluated the accuracy of the backwards difference approximation using hydrological model data:

3 $$\frac{{{\text{dTWS}}}}{{{\text{d}}t}} \approx \frac{{{\text{TWSA}}_{t} - {\text{TWSA}}_{t - 1} }}{\Delta t}$$

As demonstrated in Swenson and Wahr (2006a) and as illustrated in Fig. 6b, approximation errors for a given month can be very large, extreme values are generally underestimated and may also be temporally shifted. Users should very carefully take these approximation errors into account when deriving any conclusions about water balance closure or phase shifts between water storage and water fluxes on a monthly basis. Because GRACE observations also contain noise, computing centred differences has been reported to provide a more robust and less noisy approximation of $\text{dTWS}/dt$ as compared to forwards or backwards differences (Landerer et al. 2010; Long et al. 2014; Pascolini-Campbell et al. 2020). This means computing the difference between the next and the preceding months and dividing that by the time difference. Note that this introduces some smoothing in the estimate of $\text{dTWS}/dt$ (see Fig. 6b).

4 $$\frac{{{\text{dTWS}}}}{{{\text{d}}t}} \approx \frac{{{\text{TWSA}}_{t + 1} - {\text{TWSA}}_{t - 1} }}{2\Delta t}$$

The most important takeaway here is that computing a derivative of monthly TWS to obtain monthly TWS changes ($\Delta$ TWS, or $\Delta {\text{TWSA}}_{m}$) does *not* provide a necessarily accurate estimate of $\text{dTWS}/dt$ for use in Eq. 1 (Rodell et al. 2004; Swenson and Wahr 2006a).

If daily estimates of water fluxes (i.e. of P-ET-R) are available, it becomes possible to more accurately compare GRACE monthly TWS data against water fluxes (Rodell et al. 2004). In the following, we provide equations that can support such a formal comparison between water fluxes and monthly GRACE data. First, TWS is defined as the average TWS calculated over a whole month *m* which includes several days *d* = *[1,…, n]*:

5 $${\text{TWS}}_{m} = \frac{1}{n}\mathop \sum \limits_{d = 1}^{n} {\text{TWS}}_{d}$$

Because GRACE can only provide anomalies with respect to some unknown long-term TWS average, what is actually measured is a TWS anomaly (TWSA):

6 $${\text{TWSA}}_{m} = {\text{TWS}}_{m} - \overline{{{\text{TWS}}}} = \frac{1}{n}\mathop \sum \limits_{d = 1}^{n} {\text{TWS}}_{d} - \overline{{{\text{TWS}}}}$$

$\text{TW{S}}_{d}$ is the absolute water storage on a given day of the month. (This absolute quantity cannot be derived from GRACE observations, as the satellites are affected by all mass, not just water mass.) Neglecting lateral water redistribution, $\text{TWS}_{d}$ can be defined as the accumulation of the water fluxes since the start of the month, plus the TWS amount which was already there just before the month started (Rodell et al. 2004; Swenson and Wahr 2006a). For instance, with P_i_, ET_i_, and R_i_ expressing daily sums,

7 $${\text{TWS}}_{d} = \left[ {\sum\limits_{i = 1}^{d} {P_{i} } - {\text{ET}}_{i} - R_{i} } \right] + {\text{TWS}}_{d = 0}$$

Replacing TWS_d_ in Eq. 6 with the above expression yields the following

8 $${\text{TWSA}}_{m} = \frac{1}{n}\mathop \sum \limits_{d = 1}^{n} \left[ {\mathop \sum \limits_{i = 1}^{d} P_{i} - {\text{ET}}_{i} - R_{i} } \right] + {\text{TWS}}_{d = 0} - \overline{{{\text{TWS}}}}$$

This equation shows that the GRACE TWS anomaly of a given month is related to the mean of the cumulative sums of the water fluxes for each day of the month, plus two unknown offsets. These offsets conveniently cancel out with differentiation. For instance, using Eq. 8 and computing the backwards difference between two consecutive GRACE months m_12_ and m_34,_ which start at days n_1_ and n_3_, and end at days n_2_ and n_4_, respectively, we obtain (neglecting measurement errors):

9 $$\begin{aligned} \frac{{{\text{dTWS}}A}}{dt} = & \frac{{{\text{TWSA}}_{{m_{34} }} - {\text{TWSA}}_{{m_{12} }} }}{\Delta t} \\ = & \frac{\begin{gathered} \frac{1}{{\left( {n_{4} - n_{3} + 1} \right)}}\mathop \sum \nolimits_{{d = n_{3} }}^{{n_{4} }} \left[ {\mathop \sum \nolimits_{{i = n_{1} }}^{d} P_{i} - {\text{ET}}_{i} - R_{i} } \right] - \frac{1}{{\left( {n_{2} - n_{1} + 1} \right)}}\mathop \sum \nolimits_{{d = n_{1} }}^{{n_{2} }} \left[ {\mathop \sum \nolimits_{{i = n_{1} }}^{d} P_{i} - {\text{ET}}_{i} - R_{i} } \right] \hfill \\ \end{gathered} }{\Delta t} \\ \end{aligned}$$

where $\Delta t$ is the time difference between the middle points of the two months. This formulation corresponds to the difference in the running means of the flux accumulations (Rodell et al. 2011). Provided daily estimates of P, ET, and R, are available, TWSA differences can be formally compared to water flux variables using Eq. 9.

### Aggregating errors

When interpreting user-level gridded GRACE data, it is best to assume that neighbouring pixels are not independent of each other. This needs to be taken into account when computing error estimates for the TWS average at the basin scale or region scale (Bevington and Robinson 2003). When computing an average over an area containing $i=[1,\dots ,n]$ pixels, the aggregated error variance ${\sigma }^{2}$ is obtained as the sum of the variance–covariance matrix of all the (grid point level) errors.

10 $$\sigma^{2} = \mathop \sum \limits_{i = 1}^{n} \mathop \sum \limits_{j = 1}^{n} w_{i} \sigma_{i} \cdot w_{j} \sigma_{j} \cdot \rho_{i,j}$$

where $w$ is the weight assigned to each grid point (for an arithmetic average, $w=1/n$) and ${\rho }_{i,j}$ is the correlation *between the errors (not between the TWS time series)* at the grid level. That correlation is assumed to decay exponentially as a function of the (spherical) distance between grid points ${D}_{i,j}$, with a rate that is conditioned by the so-called de-correlation length ($l$) of the error. (Note that this isotropic approach neglects the north–south error structure induced by stripes.) The GRC Tellus Land webpage^1^ recommends to use $l=300$ km for the measurement error and $l=100$ km for the leakage error. It also provides a pseudo-code for implementing this equation.

11 $$\rho_{i,j} = e^{{\frac{{ - \left( {D_{i,j} } \right)^{2} }}{{2l^{2} }} }}$$

The total error is usually estimated in quadrature (i.e. assuming the measurement and leakage errors are independent): ${\sigma }_{\mathrm{total}}^{2}={\sigma }_{\mathrm{measurement}}^{2}+{\sigma }_{\mathrm{leakage}}^{2}$

For the 3° JPL mascons, assuming that the measurement errors between mascons are independent, the above formulation can be adapted to ${\rho }_{i,j}=1$ if grid points i and j belong to the same mascon and ${\rho }_{i,j}=0$ otherwise (for the measurement errors only). Other approaches to determine regional uncertainties from mascon products are discussed in Loomis et al. (2019).

## Applications of GRACE in Hydrology and Climate Science

This section provides only a brief overview of some of the most common applications of space-based terrestrial water storage observations for freshwater resources. For a more complete perspective, we also suggest the reviews by Frappart and Ramillien (2018) and Chen et al. (2016) for groundwater monitoring, Rodell et al. (2018) for long-term trends in freshwater resources, Girotto and Rodell (2019) for extremes in terrestrial water storage, Humphrey et al. (2016) for a climatological summary, Li et al. (2019) for data assimilation into numerical hydrological models, Tapley et al. (2019) for climate change impacts detection with GRACE, and Chen et al. (2022) for a broader review including GRACE Follow-On.

### Budget Residual Approaches

Because TWS is linked to other components of the water cycle (Eq. 1 and 2), it can be used in combination with other well-observed components in order to estimate another (less well-observed) term as the residual of the budget equation. For instance, using Eq. 2, it is possible to estimate groundwater storage changes as ΔGW = ΔTWS – ΔSM – ΔSWE – ΔSW – ΔLI – ΔBW, provided reliable estimates of all the other components are available. For instance, Rodell et al. (2007) applied this approach to estimate groundwater changes in the Mississippi basin using GRACE TWS observations, as well as hydrological model estimates of soil moisture and snow water equivalent (other components were sufficiently small to be neglected). These basin-scale estimates were then compared against in situ groundwater well observations. This type of approach has been successfully repeated to monitor seasonal as well as long-term groundwater changes over many different regions and aquifers of the world (Yeh et al. 2006; Rodell et al. 2009; Famiglietti et al. 2011; Shamsudduha et al. 2012; Feng et al. 2013; Richey et al. 2015a), and requires a careful consideration of the error propagation. Indeed, because ΔGW is estimated as the residual of the other terms, it also accumulates estimation errors from all these variables. In combination with other data, GRACE-based groundwater variations can be used to identify unsustainable depletion of water resources (Richey et al. 2015b).

Another type of budget approach is to invert Eq. 1 in order to estimate water fluxes in a way that gives proper consideration to the particular nature of GRACE measurements (see Sect. 3.5*Integration with the water budget*). Because evapotranspiration is arguably one of the most ill-observed water cycle variables, there is great interest in obtaining large-scale estimates using a combination of GRACE TWS, precipitation, and runoff data (Rodell et al. 2004; Swenson and Wahr 2006a; Long et al. 2014). Recently, this approach has been used to evaluate model-based evapotranspiration over the Amazon basin (Swann and Koven 2017), and U.S. basins (Pascolini-Campbell et al. 2020). Other authors have used the same approach to estimate other components of the water balance equation. For instance, Behrangi et al. (2017) or Girotto et al. (2021) have used GRACE data to constrain or improve precipitation estimates, with the most success for snowfall. Freshwater discharge has also been estimated from GRACE using a budget approach for instance in Syed et al. (2005) or in Famiglietti et al. (2009).

### Monitoring Extremes

Unlike precipitation-based or meteorological drought indices, GRACE directly observes changes in available freshwater resources, thus providing particularly useful information for hydrological drought monitoring. Many different characteristics of large-scale hydrological droughts such as duration, peak magnitude, severity, or spatial extent have been investigated with GRACE data. A large number of drought indices or metrics that can be used quantify these characteristics at a regional or global scale has been proposed in previous studies (Thomas et al. 2014; Zhao et al. 2017; Kusche et al. 2016). Regionally, exceptional droughts which occurred during the GRACE record have been well studied and reported. GRACE has provided a space-based perspective of these droughts, often in the context of pre-existing freshwater scarcity and unsustainable groundwater abstractions, such as in California (Famiglietti 2014), the Colorado basin (Castle et al. 2014), Texas (Long et al. 2013), Northwestern China (Cao et al. 2015), or the Tigris-Euphrates region (Voss et al. 2013). Many of these droughts have also been studied from the perspective of decadal climate variability and oscillations like the El Niño Southern Oscillation (ENSO), for instance over the Yangtze river basin (Zhang et al. 2015), in Argentina (Chen et al. 2010b), the Amazon basin (Frappart et al. 2012; Chaudhari et al. 2019), or at the global scale (Forootan et al. 2019). In fact, most of the inter-annual variability in GRACE TWS changes can be related to climate indices such as ENSO (Ni et al. 2017; Pfeffer et al. 2021). GRACE observations are also routinely assimilated into hydrological models in order to produce continuous maps of drought indicators (Houborg et al. 2012; Li et al. 2019) and drought forecasts (Getirana et al. 2020). More recently, GRACE observations have been increasingly used as an ancillary dataset to quantify drought impacts on terrestrial ecosystems. For instance, GRACE data has been used to study the response of the Amazonian forest to the exceptional 2015–2016 drought (Yang et al. 2018; Gloor et al. 2018) or the role of groundwater in buffering the impact of heatwaves on vegetation (Mu et al. 2021). Vegetation also modulates the contribution of the different water stores to the total TWS variability (Trautmann et al. 2022). At the global and regional scales, GRACE-based terrestrial water storage variations are associated with anomalies in carbon sequestration by terrestrial ecosystems that are visible in atmospheric CO_2_ concentrations (Humphrey et al. 2018; Bastos et al. 2020).

Besides monitoring droughts, terrestrial water storage observations are also useful to monitor flood potential. Because the risk of large floods is increased in the presence of saturated soils and filled water stores, GRACE observations can be used to predict the likelihood of floods at several months’ lead time (Reager and Famiglietti 2009; Reager et al. 2014). Although they are typically more short-lived than droughts, and thus more difficult to capture in monthly GRACE solutions, major floods can also be diagnosed in the GRACE record. For instance, Chen et al. (2010a) studied the TWS anomalies associated with the exceptional 2009 Amazon flood. Over the Tonlé Sap basin, in Cambodia, Tangdamrongsub et al. (2016) could derive a remarkably robust relationship between TWS anomalies and inundated extent based on MODIS. Still, there are significantly fewer studies using GRACE to monitor floods (compared to droughts), mainly because of their limited spatial and temporal extent, which is poorly captured by the monthly and coarse resolution GRACE observations. Thus, for floods, it makes most sense to assimilate GRACE data into hydrological models with the aim of improving flood warning systems in combination with other higher-resolution data (Reager et al. 2015).

### Synergies with Hydrological and Climate Models

GRACE observations may be used in combination with numerical models in a variety of ways. Here, we will cover two broad categories. First, GRACE observations of TWS anomalies can be used as an independent benchmark to evaluate or compare a collection of hydrological and climate models. Second, GRACE data can also be assimilated into a numerical model. In this case, some information derived from GRACE is transferred to the model, with the goal of improving it. Classically, this can be done either by tuning the model parameters so that the model output best fits with GRACE data (model calibration), or by forcefully adapting the model state (and potentially breaking the mass or energy balance) so that the model is brought closer to the observations (data assimilation). Several examples of these different applications can be found in the literature.

Various internationally used model benchmarking tools rely on GRACE data to perform model intercomparisons. For instance the International Land Model Benchmarking system (ILAMB) is used to evaluate land surface and hydrological models (Collier et al. 2018), and the Earth System Model Evaluation Tool (ESMValTool) (Eyring et al. 2016b) is used to evaluate global coupled climate models participating in the Coupled Model Intercomparison Project (CMIP) (Eyring et al. 2016a). Comparisons between GRACE and hydrological models has shown that many models tend to produce a peak seasonal TWS that occurs too early compared to GRACE observations, potentially related to a general underestimation of the overall water storage capacity (Schellekens et al. 2017). Limitations in the representation of TWS between the different models (inclusion of surface and ground water stores, modelling of groundwater abstractions), as well as uncertainties in the meteorological forcing, may also explain some of the differences with GRACE observations. Consistent with these findings, land surface and hydrological models were found to lack inter-annual variability compared to GRACE, especially over semi-arid and tropical regions, a situation which further impacts the representation of the carbon cycle in these models (Humphrey et al. 2018). Models were also shown to underestimate the magnitude of long-term TWS trends compared to GRACE data (Scanlon et al. 2018; Yang et al. 2020). In a recent study, Jensen et al. (2019) compared the long-term trends in GRACE TWS against climate change induced trends in water storage as simulated by coupled climate models over the last century. Partly due to the difficulty of deriving robust trends from the short GRACE record, they found limited agreement, except in regions with strong projected drying trends such as the Mediterranean basin and the Southwestern United States. This limited agreement also occurs because a substantial fraction of the negative TWS trends observed from GRACE globally is related to groundwater abstractions rather than to climatic changes (An et al. 2021).

In addition to model evaluations, GRACE is also routinely used for calibration and/or data assimilation into hydrological models. Model parameterization exercises involving GRACE TWS include for example the work by Lo et al. (2010) who used GRACE data to calibrate groundwater parameters for the Community Land Model (CLM) or by Swenson and Lawrence (2015) who derived an optimal soil thickness map also for CLM. Werth et al. (2009) used basin-scale GRACE observations to calibrate the WaterGAP Global Hydrology Model (WGHM) and found that the model parameters which were the most sensitive to TWS as a model constraint were highly dependent on climate regions and the relative importance of the different hydrological processes (snowmelt, evapotranspiration, floodplain dynamics). In addition to studies focusing on the calibration of model parameters, a large number of studies have also used data assimilation techniques, such as Ensemble Kalman filters, with the goal improving the model’s representation of water stores and fluxes over the period where GRACE data is available, without attempting to improve model physics or parameters. In this case, assimilation of GRACE into numerical models can also be viewed as a (model-dependent) way of both downscaling and disaggregating TWS observations into constituting individual water stores (i.e. soil moisture, groundwater, etc.) (Stampoulis et al. 2019). The impact of data assimilation on the performance of hydrological models has generally been reported to be positive. For instance, Zaitchik et al. (2008) found that assimilation of GRACE TWS led to higher model skill when compared to in situ groundwater, Li et al. (2012) obtained improvements in runoff estimates in most cases, Tangdamrongsub et al. (2015) found improved performance against in situ groundwater but only slight improvement for streamflow, and Kumar et al. (2016) obtained a better performance for groundwater but regionally variable impacts in terms of river discharge and evapotranspiration. In cases where anthropogenic processes play an important role, but are not represented in the model (e.g., groundwater use and/or irrigation), assimilating GRACE data can actually lead to a significant decrease in model performance (Girotto et al. 2017). Assimilating GRACE data into numerical models also poses some critical (and not fully resolved) technical challenges because of its quite coarse spatial resolution and monthly sampling (Eicker et al. 2014). In addition, the peculiar, spatially correlated, error structures of GRACE data also need to be taken into account by the data assimilation methods (Schumacher et al. 2016; Khaki et al. 2017). Finally, assimilating only GRACE TWS data into a hydrological model may also lead to a degradation in performance for some other variables. Thus, the assimilation of GRACE simultaneously with several other remotely sensed water variables, such as microwave-based soil moisture or lake altimetry constitutes one of the logical next steps (e.g., van Dijk et al. 2014; Tian et al. 2017; Khaki et al. 2019).

## Footnotes

1 https://grace.jpl.nasa.gov/data/get-data/monthly-mass-grids-land/.

## References

[CR1] Abelen S, Seitz F (2013). Relating satellite gravimetry data to global soil moisture products via data harmonization and correlation analysis. Remote Sens Environ.

[CR2] An L, Wang J, Huang J, Pokhrel Y, Hugonnet R, Wada Y, Cáceres D, Müller Schmied H, Song C, Berthier E, Yu H, Zhang G (2021) Divergent Causes of Terrestrial Water Storage Decline Between Drylands and Humid Regions Globally. Geophys Res Lett 48. 10.1029/2021gl095035

[CR3] Bastos A, O'Sullivan M, Ciais P, Makowski D, Sitch S, Friedlingstein P, Chevallier F, Rödenbeck C, Pongratz J, Luijkx IT, Patra PK, Peylin P, Canadell JG, Lauerwald R, Li W, Smith NE, Peters W, Goll DS, Jain AK, Kato E, Lienert S, Lombardozzi DL, Haverd V, Nabel JEMS, Poulter B, Tian H, Walker AP, Zaehle S (2020) Sources of uncertainty in regional and global terrestrial CO2 exchange estimates. Glob Biogeochem Cy 34. 10.1029/2019gb006393

[CR4] Behrangi A, Gardner AS, Reager JT, Fisher JB (2017). Using GRACE to constrain precipitation amount over cold mountainous basins. Geophys Res Lett.

[CR5] Bevington PR, Robinson DK (2003) Data reduction and error analysis for the physical sciences, Third edition ed. McGraw-Hill, Boston

[CR6] Cao Y, Nan Z, Cheng G (2015). GRACE Gravity satellite observations of terrestrial water storage changes for drought characterization in the arid land of Northwestern China. Remote Sens.

[CR7] Castle SL, Thomas BF, Reager JT, Rodell M, Swenson SC, Famiglietti JS (2014). Groundwater depletion during drought threatens future water security of the Colorado River Basin. Geophys Res Lett.

[CR8] Chaudhari S, Pokhrel Y, Moran E, Miguez-Macho G (2019). Multi-decadal hydrologic change and variability in the Amazon River basin: understanding terrestrial water storage variations and drought characteristics. Hydrol Earth Syst Sci.

[CR9] Chen J, Famiglietti JS, Scanlon BR, Rodell M (2016). Groundwater storage changes: present status from GRACE observations. Surv Geophys.

[CR10] Chen J, Cazenave A, Dahle C, Llovel W, Panet I, Pfeffer J, Moreira L (2022). Applications and Challenges of GRACE and GRACE follow-on satellite gravimetry. Surv Geophys.

[CR11] Chen JL, Wilson CR, Tapley BD (2010). The 2009 exceptional Amazon flood and interannual terrestrial water storage change observed by GRACE. Water Resour Res.

[CR12] Chen JL, Wilson CR, Tapley BD, Longuevergne L, Yang ZL, Scanlon BR (2010). Recent La Plata basin drought conditions observed by satellite gravimetry. J Geophys Res Atmos.

[CR13] Chen JL, Wilson CR, Li J, Zhang Z (2015). Reducing leakage error in GRACE-observed long-term ice mass change: a case study in West Antarctica. J Geodesy.

[CR14] Collier N, Hoffman FM, Lawrence DM, Keppel-Aleks G, Koven CD, Riley WJ, Mu M, Randerson JT (2018). The international land model benchmarking (ILAMB) system: design, theory, and Implementation. J Adv Model Earth Syst.

[CR15] Croteau MJ, Nerem RS, Loomis BD, Sabaka TJ (2020) Development of a daily GRACE Mascon solution for terrestrial water storage. J Geophys Res Solid Earth 125. 10.1029/2019jb018468

[CR16] Dobslaw H, Flechtner F, Bergmann-Wolf I, Dahle C, Dill R, Esselborn S, Sasgen I, Thomas M (2013). Simulating high-frequency atmosphere-ocean mass variability for dealiasing of satellite gravity observations: AOD1B RL05. J Geophys Res Oceans.

[CR17] Dobslaw H, Bergmann-Wolf I, Dill R, Poropat L, Thomas M, Dahle C, Esselborn S, König R, Flechtner F (2017). A new high-resolution model of non-tidal atmosphere and ocean mass variability for de-aliasing of satellite gravity observations: AOD1B RL06. Geophys J Int.

[CR18] Döll P, Fritsche M, Eicker A, Müller Schmied H (2014) Seasonal water storage variations as impacted by water abstractions: comparing the output of a global hydrological model with GRACE and GPS observations. Surv Geophys 35:1311–1331. 10.1007/s10712-014-9282-2

[CR19] Eicker A, Schumacher M, Kusche J, Doll P, Muller Schmied H (2014) Calibration/data assimilation approach for integrating GRACE data into the WaterGAP global hydrology model (WGHM) using an ensemble kalman filter: first results. Surv Geophys 35:1285–1309. 10.1007/s10712-014-9309-8

[CR20] Elsaka B, Raimondo J-C, Brieden P, Reubelt T, Kusche J, Flechtner F, Iran Pour S, Sneeuw N, Müller J (2013). Comparing seven candidate mission configurations for temporal gravity field retrieval through full-scale numerical simulation. J Geodesy.

[CR21] Eyring V, Bony S, Meehl GA, Senior CA, Stevens B, Stouffer RJ, Taylor KE (2016). Overview of the coupled model intercomparison project phase 6 (CMIP6) experimental design and organization. Geosci Model Dev.

[CR22] Eyring V, Righi M, Lauer A, Evaldsson M, Wenzel S, Jones C, Anav A, Andrews O, Cionni I, Davin EL, Deser C, Ehbrecht C, Friedlingstein P, Gleckler P, Gottschaldt K-D, Hagemann S, Juckes M, Kindermann S, Krasting J, Kunert D, Levine R, Loew A, Mäkelä J, Martin G, ason E, Phillips AS, Read S, Rio C, Roehrig R, Senftleben D, Sterl A, van Ulft LH, Walton J, Wang S, Williams KD (2016b) ESMValTool (v1.0)—a community diagnostic and performance metrics tool for routine evaluation of Earth system models in CMIP. Geosci Model Dev 9:1747-1802. 10.5194/gmd-9-1747-2016b

[CR23] Famiglietti JS, Syed TH, Chambers DP (2009). GRACE-based estimates of terrestrial freshwater discharge from basin to continental scales. J Hydrometeorol.

[CR24] Famiglietti JS, Lo M, Ho SL, Bethune J, Anderson KJ, Syed TH, Swenson SC, de Linage CR, Rodell M (2011). Satellites measure recent rates of groundwater depletion in California's Central Valley. Geophys Res Lett.

[CR25] Famiglietti JS (2014). The global groundwater crisis. Nat Clim Chang.

[CR26] Feng W, Zhong M, Lemoine J-M, Biancale R, Hsu H-T, Xia J (2013). Evaluation of groundwater depletion in North China using the gravity recovery and climate experiment (GRACE) data and ground-based measurements. Water Resour Res.

[CR27] Flechtner F, Reigber C, Rummel R, Balmino G (2021). Satellite gravimetry: a review of its realization. Surv Geophys.

[CR28] Forootan E, Khaki M, Schumacher M, Wulfmeyer V, Mehrnegar N, van Dijk AIJM, Brocca L, Farzaneh S, Akinluyi F, Ramillien G, Shum CK, Awange J, Mostafaie A (2019). Understanding the global hydrological droughts of 2003–2016 and their relationships with teleconnections. Sci Total Environ.

[CR29] Frappart F, Papa F, da Silva JS, Ramillien G, Prigent C, Seyler F, Calmant S (2012). Surface freshwater storage and dynamics in the Amazon basin during the 2005 exceptional drought. Environ Res Lett.

[CR30] Frappart F, Ramillien G (2018) Monitoring groundwater storage changes using the gravity recovery and climate experiment (GRACE) satellite mission: a review. Remote Sens. 10, 10.3390/rs10060829

[CR31] Getirana A, Kumar S, Girotto M, Rodell M (2017) Rivers and floodplains as key components of global terrestrial water storage variability. Geophys Res Lett 44:10359–310368. 10.1002/2017gl074684

[CR32] Getirana A, Rodell M, Kumar S, Beaudoing HK, Arsenault K, Zaitchik B, Save H, Bettadpur S (2020). GRACE improves seasonal groundwater forecast initialization over the United States. J Hydrometeorol.

[CR33] Girotto M, De Lannoy GJM, Reichle RH, Rodell M, Draper C, Bhanja SN, Mukherjee A (2017). Benefits and pitfalls of GRACE data assimilation: a case study of terrestrial water storage depletion in India. Geophys Res Lett.

[CR34] Girotto, M., and Rodell, M.: Terrestrial water storage, in: Extreme Hydroclimatic Events and Multivariate Hazards in a Changing Environment, 41–64, 2019.

[CR35] Girotto M, Reichle R, Rodell M, Maggioni V (2021) Data assimilation of terrestrial water storage observations to estimate precipitation fluxes: a synthetic experiment. Remote Sens 13. 10.3390/rs13061223

[CR36] Gloor E, Wilson C, Chipperfield MP, Chevallier F, Buermann W, Boesch H, Parker R, Somkuti P, Gatti LV, Correia C, Domingues LG, Peters W, Miller J, Deeter MN, Sullivan MJP (2018) Tropical land carbon cycle responses to 2015/16 El Niño as recorded by atmospheric greenhouse gas and remote sensing data. Philos Trans R Soc B Biol Sci 373. 10.1098/rstb.2017.0302 10.1098/rstb.2017.0302 PMC6178440 30297463

[CR37] Haagmans R, Siemes C, Massotti L, Carraz O, Silvestrin P (2020). ESA’s next-generation gravity mission concepts. Rendiconti Lincei Scienze Fisiche e Naturali.

[CR38] Han S-C, Jekeli C, Shum CK (2004) Time-variable aliasing effects of ocean tides, atmosphere, and continental water mass on monthly mean GRACE gravity field. J Geophys Res Solid Earth 109. 10.1029/2003jb002501

[CR39] Hirschi M, Seneviratne SI (2017). Basin-scale water-balance dataset (BSWB): an update. Earth Syst Sci Data.

[CR40] Houborg R, Rodell M, Li B, Reichle R, Zaitchik BF (2012). Drought indicators based on model-assimilated gravity recovery and climate experiment (GRACE) terrestrial water storage observations. Water Resour Res.

[CR41] Humphrey V, Gudmundsson L, Seneviratne SI (2016). Assessing global water storage variability from GRACE: trends, seasonal cycle, subseasonal anomalies and extremes. Surv Geophys.

[CR42] Humphrey V, Zscheischler J, Ciais P, Gudmundsson L, Sitch S, Seneviratne SI (2018). Sensitivity of atmospheric CO2 growth rate to observed changes in terrestrial water storage. Nature.

[CR43] Humphrey V, Gudmundsson L (2019). GRACE-REC: a reconstruction of climate-driven water storage changes over the last century. Earth Syst Sci Data.

[CR44] Jensen L, Eicker A, Dobslaw H, Stacke T, Humphrey V (2019). Long-term wetting and drying trends in land water storage derived from GRACE and CMIP5 models. J Geophys Res Atmos.

[CR45] Khaki M, Schumacher M, Forootan E, Kuhn M, Awange JL, van Dijk AIJM (2017). Accounting for spatial correlation errors in the assimilation of GRACE into hydrological models through localization. Adv Water Resour.

[CR46] Khaki M, Hoteit I, Kuhn M, Forootan E, Awange J (2019). Assessing data assimilation frameworks for using multi-mission satellite products in a hydrological context. Sci Total Environ.

[CR47] Klees R, Zapreeva EA, Winsemius HC, Savenije HHG (2007). The bias in GRACE estimates of continental water storage variations. Hydrol Earth Syst Sci.

[CR48] Kumar SV, Zaitchik BF, Peters-Lidard CD, Rodell M, Reichle R, Li B, Jasinski M, Mocko D, Getirana A, De Lannoy G, Cosh MH, Hain CR, Anderson M, Arsenault KR, Xia Y, Ek M (2016). Assimilation of gridded GRACE terrestrial water storage estimates in the North American land data assimilation system. J Hydrometeorol.

[CR49] Kurtenbach E, Eicker A, Mayer-Gürr T, Holschneider M, Hayn M, Fuhrmann M, Kusche J (2012). Improved daily GRACE gravity field solutions using a Kalman smoother. J Geodyn.

[CR50] Kusche J (2007). Approximate decorrelation and non-isotropic smoothing of time-variable GRACE-type gravity field models. J Geodesy.

[CR51] Kusche J, Eicker A, Forootan E, Springer A, Longuevergne L (2016). Mapping probabilities of extreme continental water storage changes from space gravimetry. Geophys Res Lett.

[CR52] Lambert A, Huang J, van der Kamp G, Henton J, Mazzotti S, James TS, Courtier N, Barr AG (2013). Measuring water accumulation rates using GRACE data in areas experiencing glacial isostatic adjustment: The Nelson River basin. Geophys Res Lett.

[CR53] Landerer FW, Dickey JO, Güntner A (2010) Terrestrial water budget of the Eurasian pan-Arctic from GRACE satellite measurements during 2003–2009. J Geophys Res 115. 10.1029/2010jd014584

[CR54] Landerer FW, Swenson SC (2012). Accuracy of scaled GRACE terrestrial water storage estimates. Water Resour Res.

[CR55] Landerer FW, Flechtner FM, Save H, Webb FH, Bandikova T, Bertiger WI, Bettadpur SV, Byun SH, Dahle C, Dobslaw H, Fahnestock E, Harvey N, Kang Z, Kruizinga GLH, Loomis BD, McCullough C, Murböck M, Nagel P, Paik M, Pie N, Poole S, Strekalov D, Tamisiea ME, Wang F, Watkins MM, Wen HY, Wiese DN, Yuan DN (2020) Extending the global mass change data record: GRACE follow‐on instrument and science data performance. Geophys Res Lett 47. 10.1029/2020gl088306

[CR56] Levine PA, Randerson JT, Chen Y, Pritchard MS, Xu M, Hoffman FM (2019). Soil Moisture variability intensifies and prolongs Eastern Amazon temperature and carbon cycle response to El Niño-Southern oscillation. J Clim.

[CR57] Li B, Rodell M, Zaitchik BF, Reichle RH, Koster RD, van Dam TM (2012). Assimilation of GRACE terrestrial water storage into a land surface model: Evaluation and potential value for drought monitoring in western and central Europe. J Hydrol.

[CR58] Li B, Rodell M, Kumar S, Beaudoing HK, Getirana A, Zaitchik BF, Goncalves LG, Cossetin C, Bhanja S, Mukherjee A, Tian S, Tangdamrongsub N, Long D, Nanteza J, Lee J, Policelli F, Goni IB, Daira D, Bila M, Lannoy G, Mocko D, Steele-Dunne SC, Save H, Bettadpur S (2019). Global GRACE data assimilation for groundwater and drought monitoring: advances and challenges. Water Resour Res.

[CR59] Li F, Kusche J, Chao N, Wang Z, Löcher A (2021) Long‐term (1979‐present) total water storage anomalies over the global land derived by reconstructing GRACE data. Geophys Res Lett 48. 10.1029/2021gl093492

[CR60] Lo MH, Famiglietti JS, Yeh PJF, Syed TH (2010) Improving parameter estimation and water table depth simulation in a land surface model using GRACE water storage and estimated base flow data. Water Resources Res 46

[CR61] Löcher A, Kusche J (2020) A hybrid approach for recovering high-resolution temporal gravity fields from satellite laser ranging. J Geodesy 95. 10.1007/s00190-020-01460-x

[CR62] Long D, Scanlon BR, Longuevergne L, Sun AY, Fernando DN, Save H (2013). GRACE satellite monitoring of large depletion in water storage in response to the 2011 drought in Texas. Geophys Res Lett.

[CR63] Long D, Longuevergne L, Scanlon BR (2014). Uncertainty in evapotranspiration from land surface modeling, remote sensing, and GRACE satellites. Water Resour Res.

[CR64] Long D, Longuevergne L, Scanlon BR (2015). Global analysis of approaches for deriving total water storage changes from GRACE satellites. Water Resour Res.

[CR65] Loomis BD, Luthcke SB, Sabaka TJ (2019). Regularization and error characterization of GRACE mascons. J Geodesy.

[CR66] Loomis BD, Rachlin KE, Wiese DN, Landerer FW, Luthcke SB (2020) Replacing GRACE/GRACE‐FO with satellite laser ranging: impacts on antarctic ice sheet mass change. Geophys Res Lett 47. 10.1029/2019gl085488

[CR67] Luthcke SB, Zwally HJ, Abdalati W, Rowlands DD, Ray RD, Nerem RS, Lemoine FG, McCarthy JJ, Chinn DS (2006). Recent Greenland ice mass loss by drainage system from satellite gravity observations. Science.

[CR68] Mu M, De Kauwe MG, Ukkola AM, Pitman AJ, Guo W, Hobeichi S, Briggs PR (2021). Exploring how groundwater buffers the influence of heatwaves on vegetation function during multi-year droughts. Earth System Dynamics.

[CR69] Ni S, Chen J, Wilson CR, Li J, Hu X, Fu R (2017). Global terrestrial water storage changes and connections to ENSO events. Surv Geophys.

[CR70] Oki T, Musiake K, Matsuyama H, Masuda K (1995). Global atmospheric water balance and runoff from large river basins. Hydrol Process.

[CR71] Pail R, Bingham R, Braitenberg C, Dobslaw H, Eicker A, Güntner A, Horwath M, Ivins E, Longuevergne L, Panet I, Wouters B (2015). Science and user needs for observing global mass transport to understand global change and to benefit society. Surv Geophys.

[CR72] Pascolini-Campbell MA, Reager JT, Fisher JB (2020) GRACE-based mass conservation as a validation target for basin‐scale evapotranspiration in the contiguous United States. Water Resources Res 56. 10.1029/2019wr026594

[CR73] Peidou A, Pagiatakis S (2020) Stripe Mystery in GRACE Geopotential Models Revealed. Geophys Res Lett 47. 10.1029/2019gl085497

[CR74] Peltier WR, Argus DF, Drummond R (2015). Space geodesy constrains ice age terminal deglaciation: the global ICE-6G_C (VM5a) model. J Geophys Res-Sol Ea.

[CR75] Pfeffer J, Cazenave A, Barnoud A (2021). Analysis of the interannual variability in satellite gravity solutions: detection of climate modes fingerprints in water mass displacements across continents and oceans. Clim Dyn.

[CR76] Reager J, Thomas A, Sproles E, Rodell M, Beaudoing H, Li B, Famiglietti J (2015). Assimilation of GRACE terrestrial water storage observations into a land surface model for the assessment of regional flood potential. Remote Sensing.

[CR77] Reager JT, Famiglietti JS (2009) Global terrestrial water storage capacity and flood potential using GRACE. Geophys Res Lett 36. 10.1029/2009gl040826

[CR78] Reager JT, Thomas BF, Famiglietti JS (2014). River basin flood potential inferred using GRACE gravity observations at several months lead time. Nat Geosci.

[CR79] Reager JT, Gardner AS, Famiglietti JS, Wiese DN, Eicker A, Lo MH (2016). A decade of sea level rise slowed by climate-driven hydrology. Science.

[CR80] Richey AS, Thomas BF, Lo M-H, Famiglietti JS, Swenson S, Rodell M (2015). Uncertainty in global groundwater storage estimates in a Total Groundwater Stress framework. Water Resour Res.

[CR81] Richey AS, Thomas BF, Lo M-H, Reager JT, Famiglietti JS, Voss K, Swenson S, Rodell M (2015). Quantifying renewable groundwater stress with GRACE. Water Resour Res.

[CR82] Rodell M, Famiglietti JS (2001). An analysis of terrestrial water storage variations in Illinois with implications for the Gravity Recovery and Climate Experiment (GRACE). Water Resour Res.

[CR83] Rodell M, Famiglietti JS, Chen J, Seneviratne SI, Viterbo P, Holl S, Wilson CR (2004). Basin scale estimates of evapotranspiration using GRACE and other observations. Geophys Res Lett.

[CR84] Rodell M, Chao BF, Au AY, Kimball JS, McDonald KC (2005). Global biomass variation and its geodynamic effects: 1982–98. Earth Interact.

[CR85] Rodell M, Chen J, Kato H, Famiglietti JS, Nigro J, Wilson CR (2007). Estimating groundwater storage changes in the Mississippi River basin (USA) using GRACE. Hydrogeol J.

[CR86] Rodell M, Velicogna I, Famiglietti JS (2009). Satellite-based estimates of groundwater depletion in India. Nature.

[CR87] Rodell M, McWilliams EB, Famiglietti JS, Beaudoing HK, Nigro J (2011). Estimating evapotranspiration using an observation based terrestrial water budget. Hydrol Process.

[CR88] Rodell M, Famiglietti JS, Wiese DN, Reager JT, Beaudoing HK, Landerer FW, Lo MH (2018). Emerging trends in global freshwater availability. Nature.

[CR89] Rowlands DD, Luthcke SB, Klosko SM, Lemoine FGR, Chinn DS, McCarthy JJ, Cox CM, Anderson OB (2005) Resolving mass flux at high spatial and temporal resolution using GRACE intersatellite measurements. Geophys Res Lett 32: n/a-n/a. 10.1029/2004gl021908

[CR90] Rowlands DD, Luthcke SB, McCarthy JJ, Klosko SM, Chinn DS, Lemoine FG, Boy JP, Sabaka TJ (2010) Global mass flux solutions from GRACE: a comparison of parameter estimation strategies—mass concentrations versus Stokes coefficients. J Geophys Res 115. 10.1029/2009jb006546

[CR91] Save H, Bettadpur S, Tapley BD (2012). Reducing errors in the GRACE gravity solutions using regularization. J Geodesy.

[CR92] Save H, Bettadpur S, Tapley BD (2016). High-resolution CSR GRACE RL05 mascons. J Geophys Res Solid Earth.

[CR93] Scanlon BR, Zhang Z, Save H, Wiese DN, Landerer FW, Long D, Longuevergne L, Chen J (2016). Global evaluation of new GRACE mascon products for hydrologic applications. Water Resour Res.

[CR94] Scanlon BR, Zhang Z, Save H, Sun AY, Müller Schmied H, van Beek LPH, Wiese DN, Wada Y, Long D, Reedy RC, Longuevergne L, Döll P, Bierkens MFP (2018). Global models underestimate large decadal declining and rising water storage trends relative to GRACE satellite data. Proc Natl Acad Sci.

[CR95] Schellekens J, Dutra E, Martínez-de la Torre A, Balsamo G, van Dijk A, Sperna Weiland F, Minvielle M, Calvet J-C, Decharme B, Eisner S, Fink G, Flörke M, Peßenteiner S, van Beek R, Polcher J, Beck H, Orth R, Calton B, Burke S, Dorigo W, Weedon GP (2017) A global water resources ensemble of hydrological models: the eartH2Observe Tier-1 dataset. Earth Syst Sci Data 9:389-413. 10.5194/essd-9-389-2017

[CR96] Schmidt R, Schwintzer P, Flechtner F, Reigber C, Güntner A, Döll P, Ramillien G, Cazenave A, Petrovic S, Jochmann H, Wünsch J (2006). GRACE observations of changes in continental water storage. Global Planet Change.

[CR97] Schumacher M, Kusche J, Doll P (2016). A systematic impact assessment of GRACE error correlation on data assimilation in hydrological models. J Geodesy.

[CR98] Seo K-W, Wilson CR, Chen J, Waliser DE (2008). GRACE's spatial aliasing error. Geophys J Int.

[CR99] Shamsudduha M, Taylor RG, Longuevergne L (2012) Monitoring groundwater storage changes in the highly seasonal humid tropics: Validation of GRACE measurements in the Bengal Basin. Water Resources Res 48. 10.1029/2011wr010993

[CR100] Stampoulis D, Reager JT, David CH, Andreadis KM, Famiglietti JS, Farr TG, Trangsrud AR, Basilio RR, Sabo JL, Osterman GB, Lundgren PR, Liu Z (2019). Model-data fusion of hydrologic simulations and GRACE terrestrial water storage observations to estimate changes in water table depth. Adv Water Resour.

[CR101] Steffen H, Petrovic S, Müller J, Schmidt R, Wünsch J, Barthelmes F, Kusche J (2009). Significance of secular trends of mass variations determined from GRACE solutions. J Geodyn.

[CR102] Swann ALS, Koven CD (2017). A Direct Estimate of the Seasonal Cycle of Evapotranspiration over the Amazon Basin. J Hydrometeorol.

[CR103] Swenson S, Wahr J (2002) Methods for inferring regional surface-mass anomalies from Gravity Recovery and Climate Experiment (GRACE) measurements of time-variable gravity. J Geophys Res Solid Earth 107:ETG 3–1-ETG 3–13. 10.1029/2001jb000576

[CR104] Swenson S, Wahr J, Milly PCD (2003) Estimated accuracies of regional water storage variations inferred from the gravity recovery and climate experiment (GRACE). Water Resources Res 39. 10.1029/2002wr001808

[CR105] Swenson S, Wahr J (2006). Estimating large-scale precipitation minus evapotranspiration from GRACE satellite gravity measurements. J Hydrometeorol.

[CR106] Swenson S, Wahr J (2006). Post-processing removal of correlated errors in GRACE data. Geophys Res Lett.

[CR107] Swenson S, Yeh PJ-F, Wahr J, Famiglietti J (2006). A comparison of terrestrial water storage variations from GRACE with in situ measurements from Illinois. Geophys Res Lett.

[CR108] Swenson S, Wahr J (2007) Multi-sensor analysis of water storage variations of the Caspian Sea. Geophys Res Lett 34. 10.1029/2007gl030733

[CR109] Swenson SC, Lawrence DM (2015). A GRACE-based assessment of interannual groundwater dynamics in the Community Land Model. Water Resour Res.

[CR110] Syed TH, Famiglietti JS, Chen J, Rodell M, Seneviratne SI, Viterbo P, Wilson CR (2005) Total basin discharge for the Amazon and Mississippi River basins from GRACE and a land-atmosphere water balance. Geophys Res Lett 32. 10.1029/2005gl024851

[CR111] Tangdamrongsub N, Steele-Dunne SC, Gunter BC, Ditmar PG, Weerts AH (2015). Data assimilation of GRACE terrestrial water storage estimates into a regional hydrological model of the Rhine River basin. Hydrol Earth Syst Sci.

[CR112] Tangdamrongsub N, Ditmar PG, Steele-Dunne SC, Gunter BC, Sutanudjaja EH (2016). Assessing total water storage and identifying flood events over Tonlé Sap basin in Cambodia using GRACE and MODIS satellite observations combined with hydrological models. Remote Sens Environ.

[CR113] Tapley BD, Bettadpur S, Ries JC, Thompson PF, Watkins MM (2004). GRACE measurements of mass variability in the Earth System. Science.

[CR114] Tapley BD, Watkins MM, Flechtner F, Reigber C, Bettadpur S, Rodell M, Sasgen I, Famiglietti JS, Landerer FW, Chambers DP, Reager JT, Gardner AS, Save H, Ivins ER, Swenson SC, Boening C, Dahle C, Wiese DN, Dobslaw H, Tamisiea ME, Velicogna I (2019). Contributions of GRACE to understanding climate change. Nat Clim Chang.

[CR115] Thomas AC, Reager JT, Famiglietti JS, Rodell M (2014). A GRACE-based water storage deficit approach for hydrological drought characterization. Geophys Res Lett.

[CR116] Tian F, Wigneron J-P, Ciais P, Chave J, Ogée J, Peñuelas J, Ræbild A, Domec J-C, Tong X, Brandt M, Mialon A, Rodriguez-Fernandez N, Tagesson T, Al-Yaari A, Kerr Y, Chen C, Myneni RB, Zhang W, Ardö J, Fensholt R (2018). Coupling of ecosystem-scale plant water storage and leaf phenology observed by satellite. Nature Ecol Evol.

[CR117] Tian S, Tregoning P, Renzullo LJ, van Dijk AIJM, Walker JP, Pauwels VRN, Allgeyer S (2017). Improved water balance component estimates through joint assimilation of GRACE water storage and SMOS soil moisture retrievals. Water Resour Res.

[CR118] Trautmann T, Koirala S, Carvalhais N, Güntner A, Jung M (2022). The importance of vegetation in understanding terrestrial water storage variations. Hydrol Earth Syst Sci.

[CR119] Tregoning P, McGirr R, Pfeffer J, Purcell A, McQueen H, Allgeyer S, McClusky SC (2022) ANU GRACE data analysis: characteristics and benefits of using irregularly shaped Mascons. J Geophys Res Solid Earth 127. 10.1029/2021jb022412

[CR120] van Dijk AIJM, Renzullo LJ, Wada Y, Tregoning P (2014). A global water cycle reanalysis (2003–2012) merging satellite gravimetry and altimetry observations with a hydrological multi-model ensemble. Hydrol Earth Syst Sci.

[CR121] Velicogna I, Wahr J (2006). Measurements of time-variable gravity show mass loss in Antarctica. Science.

[CR122] Vishwakarma B, Devaraju B, Sneeuw N (2018) What is the spatial resolution of grace satellite products for hydrology? Remote Sens. 10, 10.3390/rs10060852

[CR123] Voss KA, Famiglietti JS, Lo M, de Linage C, Rodell M, Swenson SC (2013). Groundwater depletion in the Middle East from GRACE with implications for transboundary water management in the Tigris-Euphrates-Western Iran region. Water Resour Res.

[CR124] Wahr J, Molenaar M, Bryan F (1998). Time variability of the Earth's gravity field: Hydrological and oceanic effects and their possible detection using GRACE. J Geophys Res Solid Earth.

[CR125] Wahr J, Swenson S, Zlotnicki V, Velicogna I (2004). Time-variable gravity from GRACE: First results. Geophys Res Lett.

[CR126] Wahr AGJ, Zhong S (2013) Computations of the viscoelastic response of a 3-D compressible Earth to surface loading: an application to Glacial Isostatic Adjustment in Antarctica and Canada. Geophys J Int 192:557–572. 10.1093/gji/ggs030

[CR127] Watkins MM, Wiese DN, Yuan DN, Boening C, Landerer FW (2015). Improved methods for observing Earth's time variable mass distribution with GRACE using spherical cap mascons. J Geophys Res-Sol Ea.

[CR128] Werth S, Güntner A, Petrovic S, Schmidt R (2009). Integration of GRACE mass variations into a global hydrological model. Earth Planet Sci Lett.

[CR129] Wiese DN, Nerem RS, Lemoine FG (2011). Design considerations for a dedicated gravity recovery satellite mission consisting of two pairs of satellites. J Geodesy.

[CR130] Wiese DN, Landerer FW, Watkins MM (2016). Quantifying and reducing leakage errors in the JPL RL05M GRACE mascon solution. Water Resour Res.

[CR131] Wouters B, Bonin JA, Chambers DP, Riva REM, Sasgen I, Wahr J (2014). GRACE, time-varying gravity, Earth system dynamics and climate change. Rep Prog Phys.

[CR132] Yang J, Tian H, Pan S, Chen G, Zhang B, Dangal S (2018). Amazon drought and forest response: Largely reduced forest photosynthesis but slightly increased canopy greenness during the extreme drought of 2015/2016. Glob Change Biol.

[CR133] Yang X, Tian S, Feng W, Ran J, You W, Jiang Z, Gong X (2020) Spatio-temporal evaluation of water storage trends from hydrological models over australia using grace mascon solutions. Remote Sens. 12. 10.3390/rs12213578

[CR134] Yang Y, Long D, Guan H, Scanlon BR, Simmons CT, Jiang L, Xu X (2014). GRACE satellite observed hydrological controls on interannual and seasonal variability in surface greenness over mainland Australia. J Geophys Res Biogeosci.

[CR135] Yeh PJF, Swenson SC, Famiglietti JS, Rodell M (2006) Remote sensing of groundwater storage changes in Illinois using the gravity recovery and climate experiment (GRACE). Water Resources Res 42. 10.1029/2006wr005374

[CR136] Zaitchik B, Rodell M, Reichle R (2008). Assimilation of GRACE terrestrial water storage data into a Land Surface Model: Results for the Mississippi River basin. J Hydrometeorol.

[CR137] Zhang Z, Chao BF, Chen J, Wilson CR (2015). Terrestrial water storage anomalies of Yangtze River Basin droughts observed by GRACE and connections with ENSO. Global Planet Change.

[CR138] Zhao MAG, Velicogna I, Kimball JS (2017) A global gridded dataset of grace drought severity index for 2002–14: comparison with PDSI and SPEI and a case study of the Australia Millennium Drought. J Hydrometeorol 18: 2117–2129. 10.1175/jhm-d-16-0182.1

